# Enhancing Capacitance by Increasing Electric Double Layer Disorder in Ionic Liquid Mixtures

**DOI:** 10.1021/acsnano.6c12577

**Published:** 2026-10-19

**Authors:** Hrishikesh Tupkar, Samuel G. Johnstone, Sila Alemdar, Jack McAlpine, Jack Verich, Matthew A. Gebbie

**Affiliations:** 5228 University of Wisconsin-Madison, Department of Chemical and Biological Engineering, Madison, Wisconsin 53706, United States

**Keywords:** Ionic liquids, electric double layer, surface forces, interfacial capacitance, electrostatic screening, supercapacitors

## Abstract

Supercapacitors, possessing rapid charge–discharge time scales and cycle lifetimes, provide opportunities to mitigate fluctuations from intermittent energy sources. However, while supercapacitors exhibit lower energy densities to lithium-ion batteries, advancing supercapacitor energy density is necessary to improve their utility in applications requiring rapid operating time scales. Supercapacitor energy densities critically hinge on interfacial capacitances and electrolyte voltage windows. Ionic liquids have been heavily investigated as supercapacitor electrolytes due to promising properties like high electrochemical stability, nonflammability, and nonvolatility. Yet, energy densities of ionic liquid-derived supercapacitors are often limited by low interfacial capacitances due to pronounced ion–ion correlations. Here, we show capacitance can be significantly enhanced in ionic liquids over wide voltages by mixing ionic liquids to improve electric double layer entropy. Importantly, capacitance increases exhibited by mixtures persist over the entire electrochemical stability window, showing like-charged co-ions often play similar roles to oppositely charged counterions when determining double layer capacitance. By leveraging electrochemical impedance spectroscopy and surface forces measurements, we conclude that capacitive enhancements originate from changes in short-range nanostructures in ionic liquids, where atomic force microscopy measurements reveal that mixing ionic liquids results in more compact bound ion layers with increased disorder and compressibility. Notably, we find that long-range decay lengths are statistically similar between neat ionic liquids and an equimolar mixture at potentials where we report up to 150% capacitance enhancement, indicating decoupling between the bulk electrostatic decay length and interfacial capacitance. Overall, our results reveal that modulating compositional entropy of ionic liquid double layers provides avenues to increase supercapacitor energy densities and advance next-generation energy storage.

## Graphical abstract

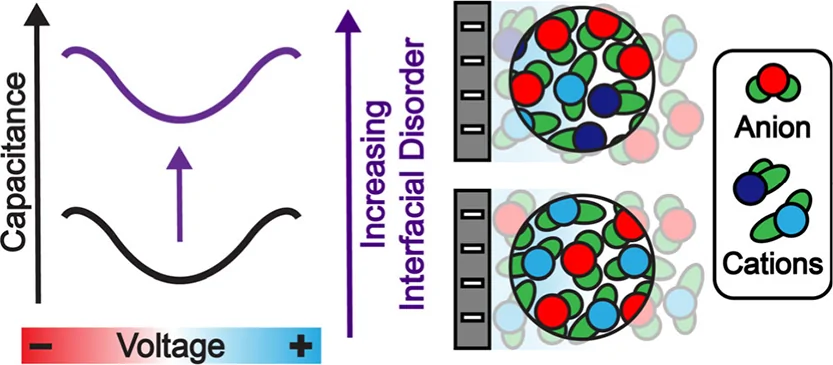

Growing energy scarcity and environmental concerns are increasingly incentivizing a global transition from fossil fuel-based energy to renewable energy sources. This wide-ranging transition has increased the implementation of intermittent energy sources such as wind and solar into applications like electric vehicles and power grids.^1–3^ This requires electrochemical energy storage devices operating on second-to-minute time scales to mitigate power fluctuations from intermittent energy. Currently, lithium-ion batteries are widely utilized for energy storage in these applications. However, while batteries exhibit high energy densities and around thousand-cycle lifetimes, using batteries to level grid-scale fluctuations is nonideal as second-to-minute time scales induce significant heating and increased risk of device failure.^4,5^

Supercapacitors, with million-cycle lifetimes and orders of magnitude higher power densities than batteries,^6–9^ are therefore garnering increasing research interest, particularly as grid electrification increases. Despite these features, supercapacitors are limited by low energy densities and improving supercapacitor energy densities can enable utilizing these devices in applications currently occupied by lower energy density batteries. The energy density of a capacitor scales with capacitance and operating voltage,^10^ as indicated in eq 1.

1 $$E=\frac{1}{2}CV^{2}$$

Here, *E* (joule) is energy, *C* (farad) is capacitance, and *V* (volt) is the voltage window of the capacitor.

Supercapacitor energy density is critically determined by the properties of the electrolyte within the device, with the ideal electrolyte possessing high capacitance over a wide operating voltage window. Problematically, current supercapacitor electrolytes are either aqueous, exhibiting limited operating voltages of 1–2 V, or contain organic solvents, which pose flammability concerns despite wider voltage windows approaching 3 V.^11–13^ Ionic liquids pose as promising electrolytes to address these limitations due to nonflammability, nonvolatility, and wide voltage windows of 3–6 V.^14–16^ Implementing ionic liquids into commercial devices requires a thorough understanding of interfacial behavior. However, high ion concentrations in ionic liquids drive the formation of ion networks via ion–ion interactions,^17^ rendering conventionally accepted models like the Gouy–Chapman–Stern model unable to predict ionic liquid capacitance. Since such models were developed for dilute electrolytes, these models assume that all ions are treated as point charges^18^ and stress that interfacial behavior is primarily dictated by counterions with co-ions largely excluded from the near-surface environment.

Increasing the fundamental understanding of interfacial behavior in ionic liquids would provide pathways toward enhancing capacitance. At a charged surface, ions within the electrolyte rearrange to compensate surface potential and the distance over which ions accumulate plays a critical role in dictating interfacial capacitance. This rearrangement forms the electric double layer,^19,20^ which consists of a short-range nanostructure of ions compensating, or screening, the surface charge in series with a longer-range diffuse layer of ions. The thickness of the diffuse layer is determined by the electrostatic decay length of the bulk electrolyte, which depends on electrolyte charge density. Classical double layer capacitance models theorize that capacitance is inversely proportional to decay length.^20,21^ These models suggest that thinner double layers result from more efficient charge compensation at the surface, which increases capacitance.

In contrast to classical dilute electrolytes where ions are fully dissociated in solvent, ions in ionic liquids are strongly correlated into charge-ordered networks.^22,23^ Given total ion concentrations in neat ionic liquids often exceed 3 M, classical theory predicts decay lengths on the order of 0.01 nm, which is much smaller than singular ion sizes. As a result, leading models that illuminate the impact of ion crowding at surfaces^24^ suggest that ionic liquids could screen charged surfaces within two bound ion layers. Yet, experiments indicate that electrostatic decay lengths of ionic liquid double layers often extend 5 to 10 nm from charged surfaces.^17,25,26^ This deviation from classical predictions occurs due to ion–ion correlations,^17,27^ which lower the effective charge concentration responding to surface potentials. Pronounced ion–ion correlations also lead to highly crowded interfaces that exhibit diminished capacitances nearing 10 μFcm^–2^.^24,28–30^ This renders ionic liquids limited for use in supercapacitors over alternatives like aqueous electrolytes, which can exhibit capacitances in the range of 20–70 μFcm^–2^.^8,31^

Such anomalous double layer thickness and capacitive behavior in ionic liquids motivates the need to mechanistically investigate how double layer structure is linked to capacitance and other electrochemical properties. Efforts to explore double layer behavior involve evaluating factors like temperature-dependence of capacitance and decay lengths, both of which are properties influenced by the double layer. For instance, increasing temperature in ionic liquids has shown to enhance capacitance^30,32,33^ and reduce decay lengths,^17,34^ suggesting that changing temperature strongly influences charge screening. Reduced decay lengths at higher temperature were attributed to disrupted ion–ion correlations and growing entropic forces,^17,35^ leading to more effective charge screening.

In this work, we systematically studied the influence of entropic forces on double layer properties by investigating ionic liquid mixtures. While prior studies have observed that ionic liquid mixtures exhibit unique charge screening properties relative to neat counterparts, many of these investigations focus on either mixed cations or mixed anions paired with an identical counterion and attribute capacitance changes to ion size or structure.^36–38^ By evaluating pairs of both cations and anions in mixtures, we aim to link changes in capacitance and double layer structure to interfacial entropy as a mechanism for modulating ionic liquid interfaces. We utilize electrochemical impedance spectroscopy (EIS) to determine the potential-dependent capacitance of mixtures varying in aromaticity, ion size, and nonpolar domains (Figure 1a). We find that the capacitance in ionic liquid mixtures is substantially dictated by both co-ions and counterions. While we find that capacitance generally increases over the entire window in mixtures relative to neat ionic liquids, our studies using amphiphilic anions reveal that capacitance enhancements can also be asymmetric within anodic and cathodic windows. Further, using a surface forces apparatus (SFA) and atomic force microscopy (AFM), we determine that capacitance is dictated within a short-range nanostructure three nanometers from a charged surface as diffuse layer decay lengths are minimally perturbed with increasing interfacial capacitance. Thus, by relating double layer thickness to capacitance, we determine the length scales that govern capacitance in ionic liquid mixtures (Figure 1b).

**Figure 1:**
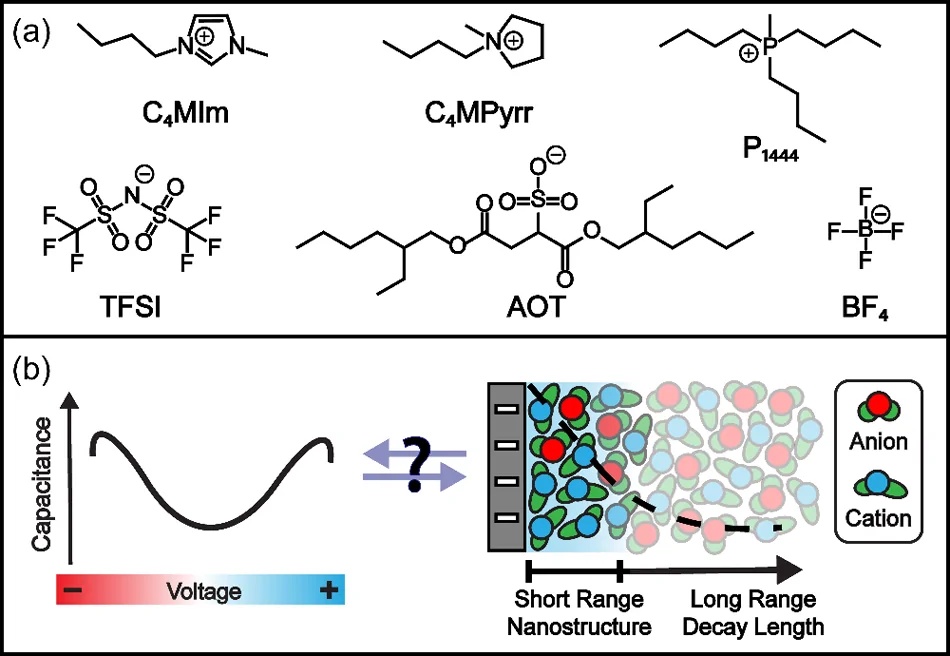
(a) Chemical structures of ionic liquid cations and anions used in this study varied by ion cores and functional groups. (b) Further exploration into ionic liquid electric double layers is needed to elucidate the length scales over which double layer formation dictates capacitance.

## Results

### Co-Ions and Counterions Together Determine Interfacial Capacitance

To understand how changes to ionic liquid structures and mixture compositions impact electric double layer capacitance, we analyzed the interfacial capacitance of ionic liquid mixtures using EIS. We collected impedance spectra at open circuit potential (OCP) within the 100 kHz to 1 Hz frequency range. Then, we increased voltage in the anodic or cathodic directions in 50 mV increments and collected impedance spectra at each potential until we reached the electrolyte stability limit. The electrolyte stability window was further studied via cyclic voltammetry (Figure S1), where current density rises sharply at stability limits. Small, spurious peaks within the electrochemical stability window are likely due to ion rearrangements and reorientation, which has been observed in ionic liquids previously.^39^ Comparing voltammetry with EIS, we find that both methods yield consistent stability windows. We represent the collected impedance spectra on Nyquist plots, which present the real and imaginary components of impedance. The shape of the impedance curve indicates the types of processes occurring within an electrochemical system, with the real components representing resistance and imaginary components corresponding to energy storage properties like capacitance.

We selected glassy carbon electrodes for our studies to ensure that measured impedance responses at the electrode-ionic liquid interface occur from ion assembly within the double layer. In contrast, metallic electrodes are known to undergo surface reconstruction and potentially even potential-induced corrosion, which impact EIS responses. Utilizing glassy carbon electrodes further extends applicability to practical supercapacitor devices, which use carbon-based electrodes. For each electrolyte tested, we observed a curved line on Nyquist representations, confirming that impedance responses are nonfaradaic and result from double layer formation (Figure S2). Curvature in low-frequency EIS is generally similar to curvature observed in the high-frequency region (Figure S3).

Using the Cole–Cole analysis,^40^ we extracted interfacial capacitance at each potential (Figure S4). We have included Nyquist representations at varying voltages to show how impedance spectra evolve with applied surface polarizations (Figure S5). Changes in impedance with applied voltage are directly reflected in capacitance values extracted from Cole–Cole analyses. We normalized capacitance values by the geometric surface area of the electrode since our goal is studying the influence of changing ionic liquid properties on double layer capacitance, and we use the same electrode for all reported experiments. The reported capacitance values are all within the electrochemical stability window of the respective electrolytes.

For analyzing capacitive responses to increasing compositional entropy, we compared neat 1-butyl-3-methylimidazolium bis­(trifluoromethylsulfonyl)­imide ([C_4_MIm]­[TFSI]) and neat 1-butyl-1-methylpyrrolidinium TFSI ([C_4_MPyrr]­[TFSI]) to ionic liquid mixtures with cation molar ratios 1:3, 1:1, and 3:1 [C_4_MPyrr]:[C_4_MIm] (Figure 2a). We chose C_4_MIm and C_4_MPyrr as cations due to similar alkyl substitution but differing chemical environments, with C_4_MIm containing an aromatic cyclic imidazolium core and C_4_MPyrr containing a nonaromatic cyclic pyrrolidinium core. We have published the capacitance of neat [C_4_MIm]­[TFSI] on glassy carbon included here previously.^41^

**Figure 2:**
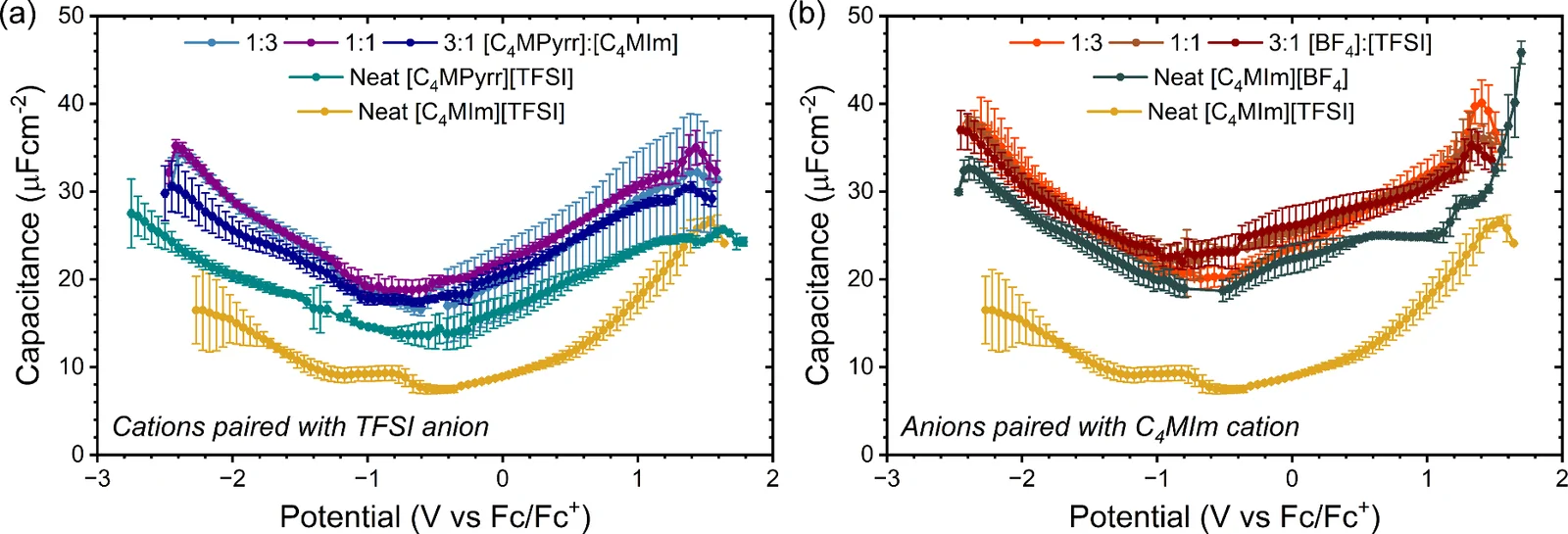
Potential-dependent capacitance of (a) [C_4_MPyrr]­[TFSI]:[C_4_MIm]­[TFSI] and (b) [C_4_MIm]­[BF_4_]:[C_4_MIm]­[TFSI] mixtures on a glassy carbon surface. All mixtures exhibit a concentration-independent increase in capacitance over the entire voltage window.

In the neat ionic liquids, we observe that [C_4_MPyrr]­[TFSI] exhibits an average of 59% higher capacitance than neat [C_4_MIm]­[TFSI] from −2.27 to 1.34 V vs Fc/Fc^+^. This difference in capacitance is consistent with recent molecular dynamics simulations comparing C_4_MPyrr and C_4_MIm ionic liquids at graphene surfaces, which indicated differences in cation adsorption geometries, with C_4_MPyrr cations orienting parallel to the electrode and C_4_MIm cations generally assembling at a tilted orientation.^42^ Hence, we conclude that observed changes in capacitance can originate from changes in near surface cation ordering. At anodic polarizations more positive than 1.34 V, we observe that the neat ionic liquids approach equivalent magnitudes of capacitance. While the anodic stability of both ionic liquids is similar, the cathodic stability of [C_4_MPyrr]­[TFSI] exceeds [C_4_MIm]­[TFSI] by 0.48 V. This is consistent with previous findings that pyrrolidinium-based ionic liquids are more cathodically stable than imidazolium-based ionic liquids.^14,43^

In all molar ratios of [C_4_MPyrr]­[TFSI]:[C_4_MIm]­[TFSI] mixtures, we observe a capacitive enhancement over the entire electrochemical window compared to either neat ionic liquid. At negative polarizations, the 1:1 mixture exhibits a maximum capacitance of 35.2 μFcm^–2^, which is greater than the maximum of 27.5 μFcm^–2^ in neat [C_4_MPyrr]­[TFSI] and 16.5 μFcm^–2^ in neat [C_4_MIm]­[TFSI]. The 1:3 and 3:1 mixtures resulted in similar capacitance enhancements, with mixed cation electrolytes exhibiting improved capacitance regardless of cation ratio. This suggests that mixing cations alters interfacial structure to screen the electrode potential more efficiently compared to neat ionic liquids.

Notably, we observe similar behavior at positive polarizations even though TFSI is the only counterion within the electrolytes. The 1:1 mixture exhibits a 35.0 μFcm^–2^ peak, which is higher than either neat component. Varying molar ratios of C_4_MPyrr and C_4_MIm also reached comparable peak capacitances of 32.2 μFcm^–2^ in the 1:3 mixture and 30.5 μFcm^–2^ in the 3:1 mixture. Capacitance is improved regardless of electrode potential in solutions containing identical anions. Therefore, both cations and anions significantly contribute to charge screening and capacitance at positive electrodes. Further, while the anodic stability limits of the mixtures are near each neat counterpart, the cathodic stability window is enhanced by 0.20 V relative to neat [C_4_MIm]­[TFSI]. This slight increase in stability indicates that mixtures are not necessarily limited by the least stable ionic liquid component, consistent with previous work expanding voltage windows through mixing.^44^

After studying mixed cation solutions, we repeated similar EIS measurements on solutions containing mixed anions paired with an identical cation (C_4_MIm). On glassy carbon electrodes, we evaluated mixtures of C_4_MIm tetrafluoroborate ([C_4_MIm]­[BF_4_]) and [C_4_MIm]­[TFSI] in anion molar ratios 1:3, 1:1, and 3:1 [BF_4_]:[TFSI] and compared with both neat components (Figure 2b). We chose these molar ratios for comparison with mixed cation mixtures of [C_4_MPyrr]­[TFSI] and [C_4_MIm]­[TFSI]. Neat [C_4_MIm]­[BF_4_] has a higher capacitance than [C_4_MIm]­[TFSI] over the entire electrochemical window at glassy carbon electrodes. Our measurements are consistent with prior capacitance studies of imidazolium TFSI and imidazolium BF_4_ ionic liquids at metal electrodes, where the BF_4_ ionic liquids exhibiting higher capacitance than TFSI was interpreted to originate from the smaller size and spherical geometry of BF_4_.^45,46^ Both ionic liquids have similar anodic stability windows with the cathodic stability of [C_4_MIm]­[BF_4_] exceeding [C_4_MIm]­[TFSI] by 0.20 V.

Mixed anion solutions in all molar ratios of [C_4_MIm]­[BF_4_]:[C_4_MIm]­[TFSI] exhibit higher capacitance than each neat component over entire voltage windows, resembling the behavior of mixed cation solutions. Mixtures of [C_4_MIm]­[BF_4_] and [C_4_MIm]­[TFSI] have an increased cathodic stability by 0.20 V relative to neat [C_4_MIm]­[TFSI]. As both mixed cation and mixed anion solutions enhance cathodic stability relative to neat [C_4_MIm]­[TFSI], this is further evidence toward utilizing mixing as a strategy to improve ionic liquid electrochemical stability.

Mixtures of [C_4_MPyrr]:[C_4_MIm] and [BF_4_]:[TFSI] are paired with identical counterions over the window where this capacitance enhancement is observed. Holistically, this behavior strongly suggests that both co-ions and counterions appear to contribute significantly to interfacial capacitance. Interestingly, all these mixtures contain three distinct ionic species, raising the possibility that increasing the number of ionic species in an electrolyte could increase the interfacial capacitance even further.

To evaluate how the number of different ionic species influences capacitance, we evaluated mixtures of 1:1 [C_4_MPyrr]­[TFSI]:[C_4_MIm]­[BF_4_] to compare with the mixed cation solution 1:1 [C_4_MPyrr]­[TFSI]:[C_4_MIm]­[TFSI] and the mixed anion solution 1:1 [C_4_MIm]­[BF_4_]:[C_4_MIm]­[TFSI] (Figure 3). The 1:1 [C_4_MPyrr]­[TFSI]:[C_4_MIm]­[BF_4_] mixture contains four distinct ionic species that can respond to applied electrode potential. We observe that the four-ion mixture still enhances capacitance relative to neat [C_4_MIm]­[TFSI]. However, the capacitance of this mixture matches the mixed cation or mixed anion ionic liquids at anodic polarizations. At cathodic polarizations, we observe a slight decline in capacitance with the four-ion mixture exhibiting a maximum capacitance of 34.2 μFcm^–2^ in contrast to 35.2 μFcm^–2^ in 1:1 [C_4_MPyrr]­[TFSI]:[C_4_MIm]­[TFSI] and 38.0 μFcm^–2^ in [C_4_MIm]­[BF_4_]:[C_4_MIm]­[TFSI].

**Figure 3:**
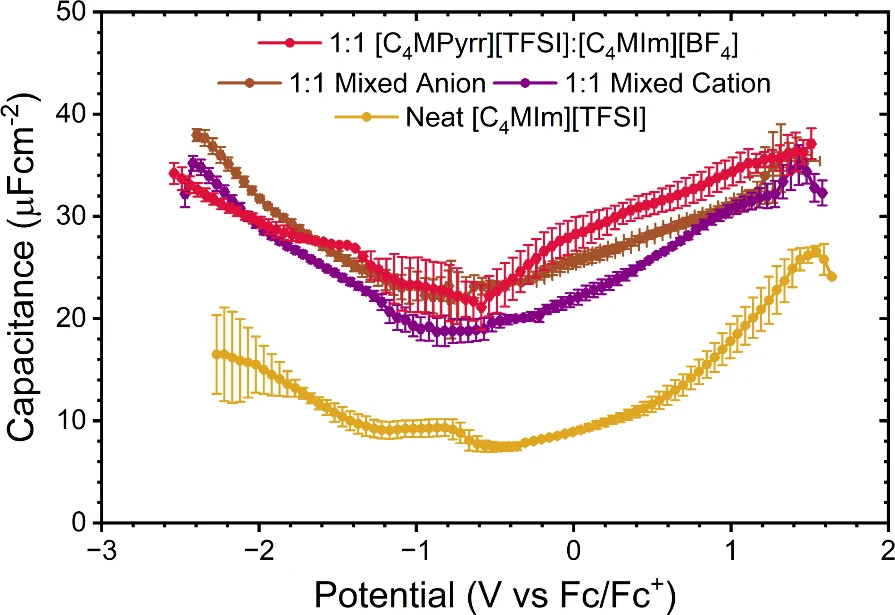
Potential-dependent capacitance of the equimolar [C_4_MPyrr]­[TFSI]:[C_4_MIm]­[BF_4_] mixture exhibits similar capacitance to 1:1 mixed cation mixtures of [C_4_MPyrr]­[TFSI]:[C_4_MIm]­[TFSI] and 1:1 mixed anion mixtures of [C_4_MIm]­[BF_4_]:[C_4_MIm]­[TFSI].

The four-ion mixture, mixed cation solution, and mixed anion solution all exhibit similar voltage windows, suggesting that adding more ionic species does not necessarily continue to improve electrochemical stability. Mixing [C_4_MPyrr]­[TFSI] with [C_4_MIm]­[BF_4_] results in electrolytes composed of the same functional groups present in the three-ion mixtures, which is likely a driving factor behind the minimal change in capacitance between three-ion and four-ion mixtures. To gain insight into how substantial changes to chemical structure can further impact electric double layer formation, we also investigated ionic liquid cations with larger nonpolar groups. The presence of larger nonpolar groups has been observed to result in self-assembly into distinct polar and nonpolar domains at charged surfaces,^31,47^ though the impact this has on interfacial capacitance remains unclear.

Investigating how larger nonpolar domains on ion structures impact interfacial capacitance, we tuned the polar-nonpolar separation within the ionic liquids. We prepared mixtures with the ionic liquid tributylmethylphosphonium (P_1444_) TFSI. P_1444_ is an aliphatic cation containing two additional nonpolar butyl chains compared to C_4_MIm and C_4_MPyrr. We performed EIS on the molar ratio 1:1:1 [P_1444_]­[TFSI]:[C_4_MPyrr]­[TFSI]:[C_4_MIm]­[TFSI] and compared with each neat ionic liquid and the 1:1 [C_4_MPyrr]:[C_4_MIm] mixture at glassy carbon electrodes (Figure 4a). We find that the 1:1:1 cation mixture containing P_1444_ exhibits a similar capacitance to 1:1 [C_4_MPyrr]­[TFSI]:[C_4_MIm]­[TFSI] from −1.12 to 0.13 V vs Fc/Fc^+^. At all other potentials, the capacitance of the 1:1:1 cation mixture is lower than 1:1 [C_4_MPyrr]:[C_4_MIm]. This indicates that the presence of P_1444_ at the electrode surface lowers interfacial capacitance relative to the 1:1 [C_4_MPyrr]:[C_4_MIm] case.

**Figure 4:**
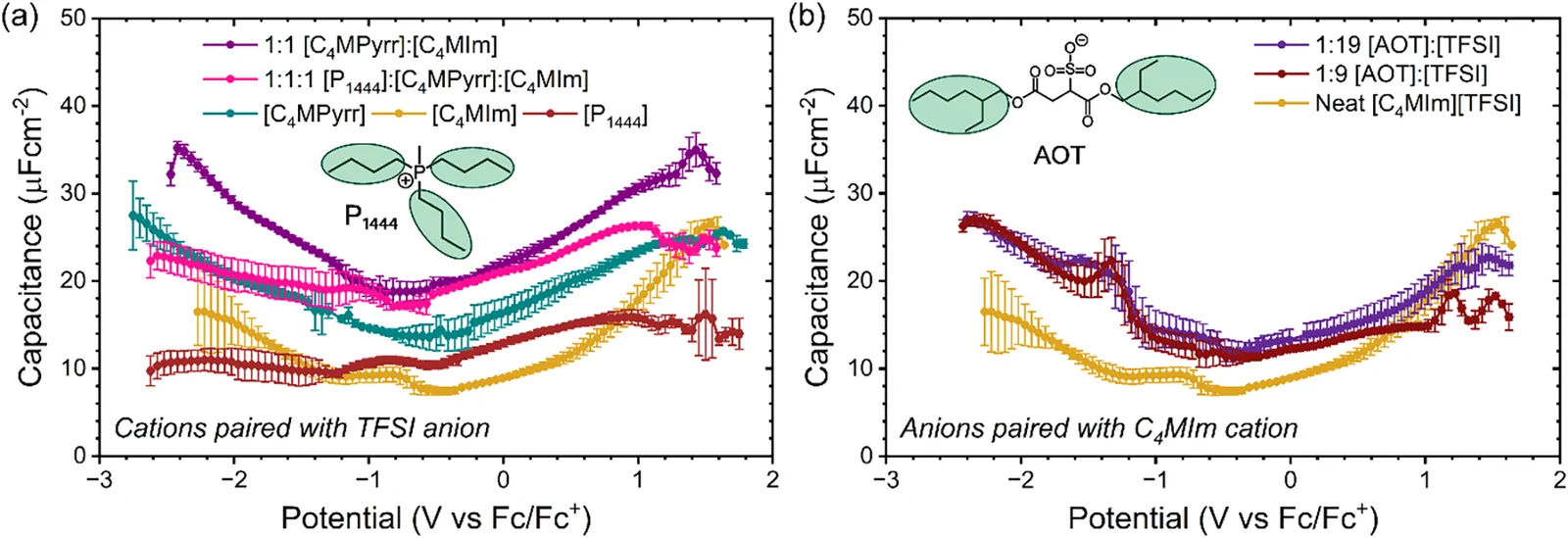
Capacitance of mixtures containing (a) 1:1:1 [P_1444_]:[C_4_MPyrr]:[C_4_MIm] paired with TFSI and (b) 1:19 and 1:9 [C_4_MIm]­[AOT]:[C_4_MIm]­[TFSI] suggest that alkyl functionality and polarity can decrease potential-dependent capacitance.

The capacitance of neat [P_1444_]­[TFSI] is lower than neat [C_4_MIm]­[TFSI] at cathodic polarizations and falls between neat [C_4_MIm]­[TFSI] and neat [C_4_MPyrr]­[TFSI] at anodic polarizations. Notably, 1:1:1 [P_1444_]:[C_4_MPyrr]:[C_4_MIm] has a higher capacitance than all three neat ionic liquids over a large potential window of −1.67 to 1.13 V vs Fc/Fc^+^. At voltages lower than −1.67 V, the capacitance of the 1:1:1 mixture matches neat [C_4_MPyrr]­[TFSI]. Similarly at positive voltages higher than 1.13 V, the 1:1:1 mixture exhibits capacitance equivalent to both neat [C_4_MPyrr]­[TFSI] and neat [C_4_MIm]­[TFSI]. Regardless, we observe that the 1:1:1 mixture of these three cations results in either increased or similar capacitances to any neat component. This highlights that the increased interfacial entropy imparted by mixing different ions appears to generally enhance capacitance over neat ionic liquids, even though the 1:1 [C_4_MPyrr]:[C_4_MIm] may exhibit higher global capacitances than the 1:1:1 mixture.

Further, the capacitance curves of the 1:1:1 mixture and neat [P_1444_]­[TFSI] appear parallel to one another, particularly at potentials where the 1:1:1 mixture capacitance is lower than the 1:1 cation mixture. This parallel behavior suggests that P_1444_ is present in the interfacial environment at these potentials and lowering capacitance. Notably, the 1:1:1 mixture is more cathodically stable than the 1:1 cation mixture by 0.15 V and neat [C_4_MIm]­[TFSI] by 0.35 V. The inclusion of [P_1444_]­[TFSI], which has a higher cathodic stability than the 1:1 mixture, improves the electrochemical stability of the electrolyte. However, the anodic stability remains unchanged relative to the 1:1 cation mixture. Here, we observe that the stability of the overall electrolyte is enhanced at the cost of lowering capacitance in the mixture. This highlights the types of technological trade-offs that must be considered when translating ionic liquid mixtures toward practical devices.

In addition to studying cations with larger nonpolar domains, we studied capacitive responses to increased polar-nonpolar separation by testing mixtures containing C_4_MIm bis­(2-ethylhexyl) sulfosuccinate (AOT). The amphiphilic anion AOT contains two nonpolar hydrocarbon chains surrounding a charged, polar group. The ionic liquid [C_4_MIm]­[AOT] has shown to form polarity-dictated nanostructures in the bulk and at the interface owing to its amphiphilic anion, and has been denoted as a surface-active ionic liquid (SAIL).^31^ At glassy carbon electrodes, we evaluated the EIS of 1:19 and 1:9 molar ratios of [C_4_MIm]­[AOT]:[C_4_MIm]­[TFSI] (Figure 4b). In mixtures, we observe that adding AOT contributes a capacitance increase at negative polarizations and decrease at positive polarizations. We report a maximum cathodic capacitance of 26.9 μFcm^–2^ in both 1:19 and 1:9 molar ratios [AOT]:[TFSI] compared to 16.5 μFcm^–2^ in neat [C_4_MIm]­[TFSI]. Anodic polarizations reveal a decline in capacitance with increasing [C_4_MIm]­[AOT] concentration.

The AOT ions form anionic bilayers in neat [C_4_MIm]­[AOT] due to favorable van der Waals interactions between the two nonpolar chains in the SAIL.^31^ This bilayer formation has been theorized to enrich the near-surface ion layers with cations, allowing more cations to pack at the surface than in nonamphiphilic ionic liquids. Although our mixtures contain more diluted concentrations of AOT, the surface activity of AOT suggests that this anion will have a significant contribution to the interfacial structure despite being present at a lower concentration than TFSI. Interestingly, we find that the addition of AOT imparts a noteworthy increase in capacitance under cathodic polarizations, highlighting the importance of co-ions in ionic liquid double layers. At the most anodic polarizations tested, the interfacial presence of AOT anions seem to lower the charge screening efficiency with increasing AOT concentration, showing a potential asymmetry in how co-ions impact interfacial capacitance as compared to counterions in ionic liquid mixtures. Nevertheless, the inclusion of AOT still results in capacitance enhancement from the capacitive minimum around −0.38 V to anodic polarizations approaching 0.82 V vs Fc/Fc^+^.

While we expect molar ratios higher than 1:9 [AOT]:[TFSI] to exhibit a further decline in anodic capacitance, we observed an oily film formed at the electrode surface upon polarization in 1:4 molar ratios [AOT]:[TFSI]. This points to an electrochemically induced demixing of the ionic liquid mixture into distinct phases due to the limited bulk solubility of [C_4_MIm]­[AOT] in [C_4_MIm]­[TFSI]. Formation of this surface film led to inconsistent results, so the capacitance data we report here is only within compositions where no film was observed over applied voltage. Regardless, we show that modulation of interaction environments plays an important role in determining the interfacial capacitance of ionic liquids at electrode surfaces.

### Long-Range Decay Lengths are Decoupled from Interfacial Capacitance in Ionic Liquids

To gain insight into how entropy of mixing impacts the bulk electrostatic decay lengths and bound ion layer structures of ionic liquids, we performed surface forces measurements and interrogated changes in the structure and extent of ionic liquid double layers. Using a miniature surface forces apparatus (μSFA),^48^ we probed the interaction forces within ionic liquid electrolytes confined between two atomically smooth mica surfaces. In contact with ionic liquids, potassium ions dissociate from mica surfaces. This results in a net negative diffuse double layer potential, previously estimated in the range of −50 to −75 mV for symmetric mica–mica electric double layer interactions across imidazolium TFSI ionic liquids.^34^ Since we conducted experiments with ionic liquids confined between two mica surfaces, symmetric electric double layers are formed in response to each negative mica surface.

Consistent with previous SFA studies, we use white light interferometry to measure the absolute separation distance between mica surfaces.^48–50^ Using a motorized micrometer, we approached the mica surfaces over a varied range of approach speeds from 1 to 3 nm/s. Over this range, we determined the surface approach velocity below which hydrodynamic interactions exhibit a negligible contribution to measured surface forces. From this analysis, we determined that the mica surface approach velocity of 3 nm/s resulted in negligible hydrodynamic coupling beginning from surface separation distances of 300 nm. Starting from surfaces around 300 nm apart, we used the micrometer to drive the surfaces into moderate contact, reaching single-nanometer separation distances where the repulsive forces applied across the surfaces approached 1 mN m^–1^. We then retracted the surfaces apart to the region with no interaction. We collected three force–distance profiles at this approach speed.

Using SFA, we collected force–distance profiles of neat [C_4_MIm]­[TFSI], neat [C_4_MPyrr]­[TFSI], and 1:1 [C_4_MPyrr]­[TFSI]:[C_4_MIm]­[TFSI], with representative force–distance profiles shown in Figure 5. The electrostatic screening length or decay length (denoted by (κ*)^−1^) of each ionic liquid electrolyte was calculated by fitting the long-range force decay using a linearized Poisson–Boltzmann equation.^34^ We chose to examine these three ionic liquids as test cases since these solutions exhibited increasing capacitance at polarizations corresponding to negative mica potential (Figure 2a). We find that the double layer potential was similar across the three ionic liquids tested. We observe only minimal angstrom-scale changes in decay lengths between the two neat ionic liquids, with the decay length being 8.9 ± 0.5 nm in [C_4_MIm]­[TFSI] and 9.2 ± 0.6 nm in [C_4_MPyrr]­[TFSI] (Figure 5). The 1:1 [C_4_MPyrr]:[C_4_MIm] mixture exhibited a decay length of 9.6 ± 0.4 nm. We determine a similar trend in SFA force runs collected using a piezoelectric motor (Figure S6).

**Figure 5:**
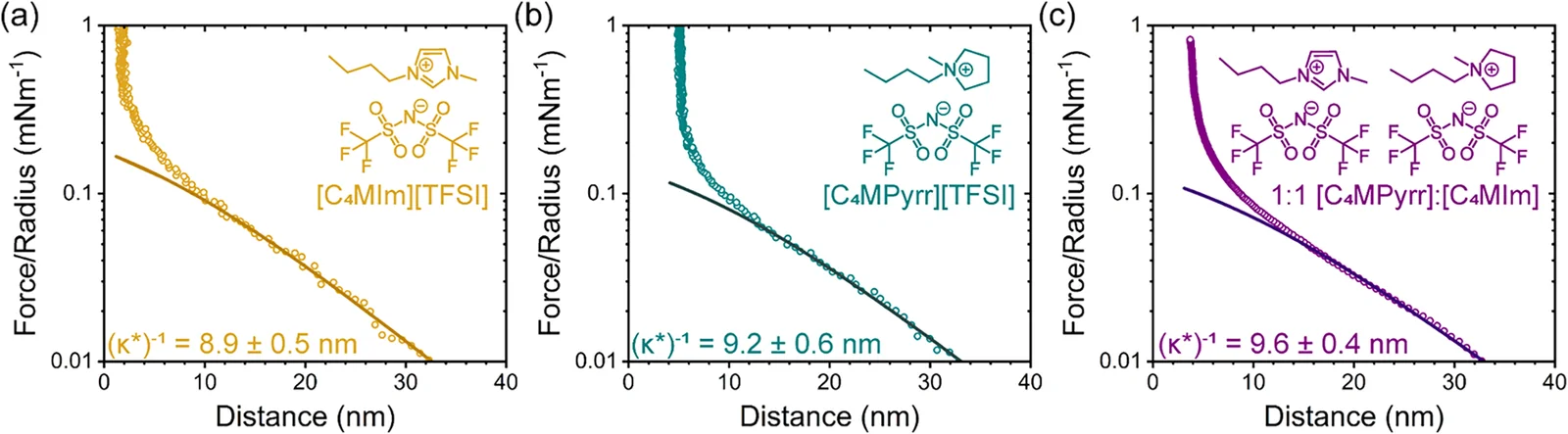
Representative force–distance profiles of (a) neat [C_4_MIm]­[TFSI], (b) neat [C_4_MPyrr]­[TFSI], and (c) 1:1 [C_4_MPyrr]­[TFSI]:[C_4_MIm]­[TFSI] illustrate an Angstrom-scale increase in decay length with multifold increase in interfacial capacitance.

Since our decay length measurements exhibit overlapping error bars, we conducted a one-way analysis of variance (ANOVA) to compute whether the decay lengths measured for each ionic liquid system are statistically different. At a 0.05 significance level, we determine that decay lengths of [C_4_MIm]­[TFSI], [C_4_MPyrr]­[TFSI], and the 1:1 [C_4_MPyrr]:[C_4_MIm] mixture are not statistically different. Therefore, all three ionic liquid systems exhibit similar long-range decay, indicating similar degrees of ionic correlations in the bulk fluids.

Since long-range decay lengths are not measurably impacted by mixing and seem to be decoupled from interfacial capacitance, we chose to only test cation mixtures as a model system. We anticipate that performing SFA measurements on other types of ionic liquid mixtures like mixed anion cases would reveal similar long-range screening behavior that is decoupled from capacitance trends.

The long-range screening length is predicted to be a function of the bulk liquid.^26^ Therefore, minimal perturbation in long-range decay with increasing interfacial capacitance suggests that capacitance is more strongly dictated by the short-range nanostructure. In addition to measuring electrostatic decay lengths, SFA enables characterization of near-surface bound ion layers as two charged surfaces are brought into molecular-scale contact. In particular, we use SFA to measure the distances over which the force curves begin to deviate from the diffuse double layer prediction and monitor the distance of closest approach. The closest approach distance that we report is the separation distance at which forces are approximately 1 mN m^–1^. By monitoring the distances of closest approach, we can evaluate whether ionic liquid mixtures induce any noteworthy change to the thicknesses or structures of surface-bound ion layers. For the three ionic liquid test cases studied, we observed that [C_4_MIm]­[TFSI] and the 1:1 [C_4_MPyrr]:[C_4_MIm] mixture exhibit similar bound ion layer thicknesses within overlapping 95% confidence intervals, with [C_4_MPyrr]­[TFSI] exhibiting a higher average bound ion layer thickness. Specifically, the length of the short-range nanostructure for each ionic liquid is half of the close approach distance as there are two surfaces involved in SFA measurements. This short-range nanostructure falls as 1.1 ± 0.2 nm in neat [C_4_MIm]­[TFSI], 2.3 ± 0.6 nm in [C_4_MPyrr]­[TFSI], and 1.3 ± 0.5 nm in the 1:1 [C_4_MPyrr]:[C_4_MIm] mixture (Table S1).

We note that we set up the measurement to be sensitive to weaker forces and accurately capture long-range diffuse layer decay, so we only approached surfaces to forces nearing 1 mN m^–1^. This rendered us less capable of performing higher compression at close approaches of the two surfaces.

### Cation Mixing Collapses Bound Ion Layers and Increases Interfacial Disorder in Ionic Liquids

To elucidate how increasing interfacial entropy changes short-range nanostructures, we employed atomic force microscopy (AFM) to determine ion ordering at charged surfaces. AFM is ideally suited to studying near-surface bound ion layers^17,51,52^ and can therefore reveal how changing ionic liquid composition influences interfacial structure. Using AFM, we measured force–distance profiles of [C_4_MIm]­[TFSI], [C_4_MPyrr]­[TFSI], and 1:1 [C_4_MPyrr]:[C_4_MIm] at mica surfaces (Figure 6). We chose mica to keep surface charge consistent with surface forces measurements conducted using SFA. For each ionic liquid system, we conducted a minimum of 150 force curves, each comprising an approach and retraction component. The approach curves are represented as heatmaps shown in Figure 6, with retraction curves provided in the Supporting Information (Figure S7). The locations of ion layers were determined using the peaks in the histograms of the apparent separation, pictured below each force–distance heatmap.

**Figure 6:**
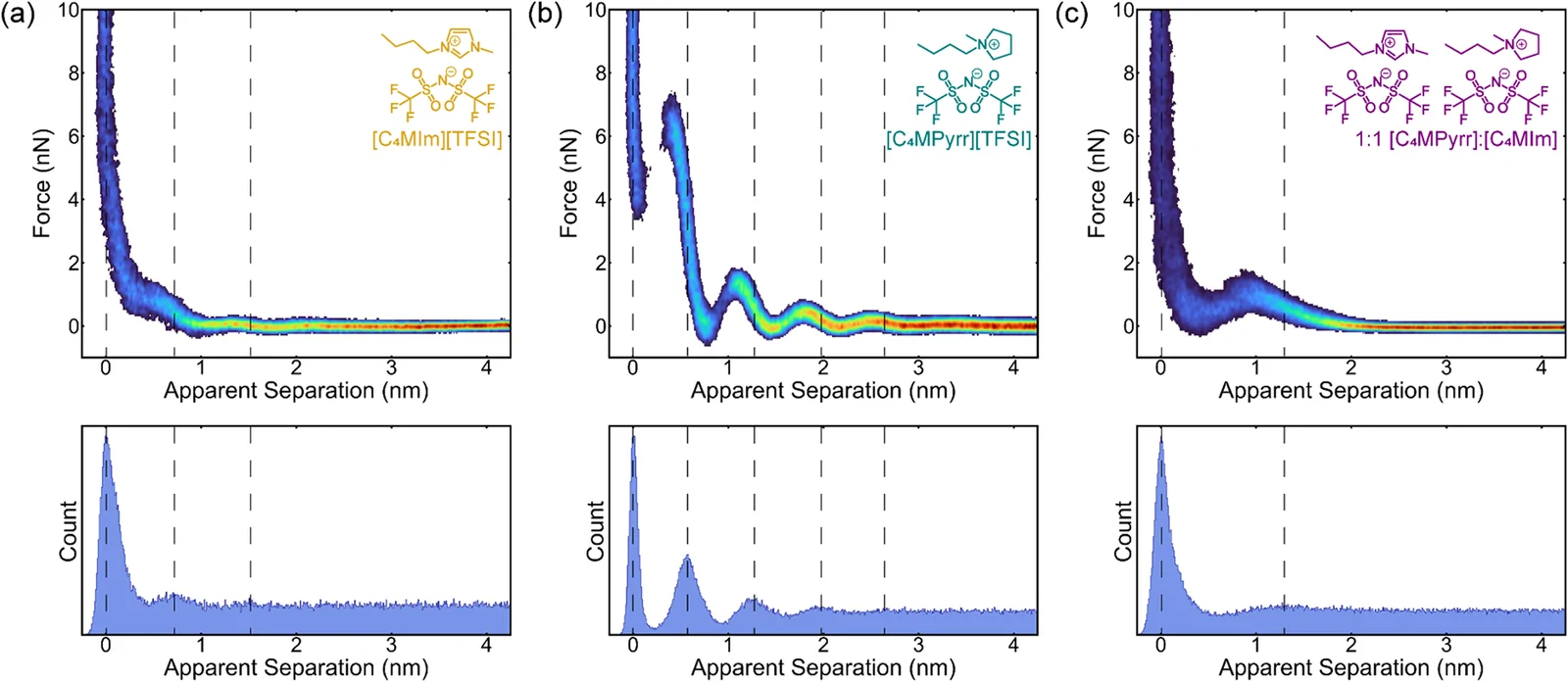
AFM force–distance heatmaps during approach for (a) [C_4_MIm]­[TFSI], (b) [C_4_MPyrr]­[TFSI], and a (c) 1:1 [C_4_MPyrr]­[TFSI]:[C_4_MIm]­[TFSI] mixture on mica. Histograms constructed from each heatmap are pictured below force–distance profiles.

In [C_4_MIm]­[TFSI], we observe 2 distinct ion layers in force heatmaps, with steps present around 0.7 and 1.5 nm from the hardwall. The force profiles of [C_4_MPyrr]­[TFSI] are much more well-defined, with the short-range nanostructure of [C_4_MPyrr]­[TFSI] revealing 4 distinct layers, with steps around 0.6, 1.2, 1.9, and 2.6 nm. The [C_4_MPyrr]­[TFSI] nanostructure is therefore more ordered and extended than the nanostructure of [C_4_MIm]­[TFSI], as illustrated in the histograms. We note that the higher degree of ordering in [C_4_MPyrr]­[TFSI] reveals that the pyrrolidinium ionic liquid is less compressible than its imidazolium counterpart.

Notably, in 1:1 [C_4_MPyrr]:[C_4_MIm], we observe that short-range nanostructure becomes condensed within a single ion layer, with a step around 1.3 nm apparent separation. The low slope in the force–distance curve at this single step indicates a softer ion layer that is more compressible than the layers observed in the neat ionic liquid systems,^53^ which is supported by an increased width of the peak in the corresponding histogram. Along with the absence of multiple distinct ion layers, this indicates the near-surface region in the mixture is more disordered than in either neat ionic liquid. Further, the histogram reveals that the mixture is not simply a superposition of the two neat ionic liquid components but rather, the mixture forms a distinct, disordered interface unique from either pure component. Therefore, with differences in ordering, compressibility, and extent of short-range nanostructures between the three ionic liquid systems, we observe that variations in short-range charge ordering poses a defining influence on interfacial capacitance.

## Discussion

By investigating the interfacial impact of compositional entropy in ionic liquid mixtures, we reveal that increases in ion composition can raise electric double layer capacitance over full voltage windows. We observe that both co-ions and counterions exhibit significant contributions to electrochemical properties. Further, we confirm that polarity-dictated nanostructure impacts double layer capacitance.^31,47^ While the dependence of capacitance on ionic liquid chemical structures has been studied previously,^29,43,54^ we focus on evaluating mixed cation and mixed anion ionic liquids to emphasize the contribution that entropy of mixing has on double layer formation. We connect these insights to a complementary investigation of long-range electrostatic screening lengths and bound ion layer thicknesses. Our work suggests that capacitance is dictated by ionic assembly occurring within three nanometers from charged surfaces, as we find that long- range decay lengths increase only slightly in ionic liquid mixtures despite significant concurrent increases in interfacial capacitance.

Notably, we see a consistent increase in the interfacial capacitance of ionic liquid mixtures relative to neat ionic liquids, highlighting how compositional entropy appears to provide an avenue to increase double layer capacitance. Thermodynamically, forming an electric double layer costs an entropic penalty, with ions demixing from the bulk electrolyte where ions are more dispersed to a more ordered arrangement at the surface.^55,56^ Reducing this penalty increases the driving force to form the double layer, improving ionic responses to an applied surface potential. Theoretical insights indicate that in correlated electrolyte systems like ionic liquids, entropy can drive mixing.^57–59^ We explore this experimentally in mixed ionic liquids, showing that entropic driving forces can be leveraged to increase electric double layer capacitance. Specifically, we leverage AFM measurements to demonstrate that ionic liquid mixtures form interfacial bound ion layers that are more disordered than either neat ionic liquid. Also, we note that the mixture exhibits a more compact nanostructure of 1.3 nm compared to 1.5 nm in neat [C_4_MIm]­[TFSI] and 2.6 nm in neat [C_4_MPyrr]­[TFSI] as determined by AFM. Therefore, these bound ion layers necessarily contain both cationic species, leading to increased interfacial entropy and increased double layer capacitance as measured via EIS. Hence, we show that the presence of more distinct ion types available to compensate surface potential increases interfacial entropy and raises capacitance in mixtures relative to neat ionic liquids.

We observe that increasing the number of anionic species in the three-ion and four-ion mixtures tends to raise interfacial capacitance across entire voltage windows with significant contribution from both counterions and co-ions (Figures 2b and 3). Capacitance in neat ionic liquids is thought to be linked to ion arrangements in the first one to two bound layers adjacent to electrode surfaces. Theoretical investigations into mixtures of BF_4_ and TFSI paired with identical imidazolium cations indicate that BF_4_ typically assembles closer to the electrode surface than TFSI due to smaller anion size and stronger cation-BF_4_ attraction.^38,44,60^ The inclusion of BF_4_ is expected to increase capacitance due to its smaller size and more compact structure, which better compensates surface potential relative to TFSI. When only considering electrostatic contributions, this inclusion would suggest that capacitance resulting from any mixtures of the two anions would fall between either neat ionic liquid. However, from our observations that capacitance is higher than either neat counterpart at cathodic and anodic polarizations, entropy of mixing must play a key role at defining interfacial structure and screening over size or compactness.

In addition to illuminating the impact of interfacial entropy on mixed anion ionic liquids, we show that the same phenomena extend to mixtures of cations C_4_MPyrr and C_4_MIm. These mixtures exhibit analogous capacitance increases across the entire voltage window when paired with the TFSI anion (Figure 2a). Hence, our results suggest that mixed cations at the interface also exhibit more efficient charge screening than either neat component. Given that mixed cations in the four-ion mixture continue to enhance capacitance relative to neat ionic liquids (Figure 3), changing interfacial entropy provides a unifying mechanism for the higher capacitance across both mixed cation and mixed anion ionic liquid systems.

To further evaluate the influence of compositional entropy on capacitance relative to ion packing and size, we added up to 10 mol percent of the amphiphilic anion AOT into [C_4_MIm]­[TFSI] (Figure 4b). According to previous understanding of ionic structures and capacitance, the addition of AOT should decrease capacitance over the entire voltage window due to its bulkier, larger size relative to TFSI. Rather, the capacitance of [C_4_MIm]­[AOT]:[C_4_MIm]­[TFSI] mixtures is higher over the entire voltage window even at mixture ratios as low as 1:19, except within the 0.8 V anodic window prior to oxidative decomposition. Hence, this result further emphasizes that interfacial entropy is a key driver of capacitance and screening even when one component is a large amphiphilic anion typically predicted to decrease capacitance due to its larger size.

Exploring the idea that capacitance can be boosted even by including larger, more nonpolar ions, we observe that the inclusion of the cation P_1444_ in 1:1:1 [P_1444_]:[C_4_MPyrr]:[C_4_MIm] mixtures raises capacitance relative to each neat component (Figure 4a). Klein et al. show that incorporating cations with varying alkyl chain lengths into ionic liquid mixtures can alter interfacial structures significantly.^36^ The P_1444_ cation has a larger proportion of nonpolar aliphatic character compared to the cyclic cations C_4_MPyrr and C_4_MIm, with the added alkyl chains on the phosphonium center increasing the overall cation size relative to cyclic cations. The trend we observe in the 1:1:1 mixture provides further validation that interfacial structures involving more ionic species exhibit an increased screening response to compensate surface charge. Therefore, despite the ionic size and polarity of the cation, increased interfacial entropy due to mixing contributes a notable capacitance increase. Also, since the voltage window of the 1:1:1 cation mixture exceeds that of the least stable component, we provide further evidence showing that ionic liquid mixtures can have higher electrochemical stability than neat ionic liquids, as observed in our studies and previous work.^61,62^

Subsequently, since increasing alkyl substituents on ions like P_1444_ and AOT increases molecule size and steric hindrance at the surface, these factors can decrease configurational entropy at the interface.^63,64^ This slight reduction in interfacial entropy could contribute to the decreased capacitance at select polarizations in the [AOT]:[TFSI] mixtures or in the 1:1:1 [P_1444_]:[C_4_MPyrr]:[C_4_MIm] mixture when compared to 1:1 [C_4_MPyrr]:[C_4_MIm] mixture (Figure 4). We observe that this thermodynamic framework holds when we vary electrode identity as well, with electric double layer formation in ionic liquid mixtures at a gold electrode exhibiting similar capacitance trends to glassy carbon (Figure S8). Regardless, we still observe an overall enhancement in capacitance compared to neat ionic liquids upon mixing, with slight variations in capacitance magnitudes owing to entropic penalties incurred from specific ion structures. Thus, compositional entropy provides a robust knob toward tuning interfacial capacitance and screening in ionic liquid mixtures.

To seek structural insight into why ionic liquid mixtures exhibit increased capacitance, we leveraged SFA to measure changes in long-range electrostatic screening length and short-range bound ion layer thicknesses. We selected neat and 1:1 mixtures of [C_4_MPyrr]­[TFSI] and [C_4_MIm]­[TFSI] confined between negative mica surfaces as a model system (Figure 5). We find that the ionic liquid mixture exhibits statistically similar measured decay lengths to either neat ionic liquid and yet, the interfacial capacitance of the mixture exceeds either neat ionic liquid. Thus, this scaling between decay length and capacitance contradicts classical dilute electrolyte theories which predict that increases in double layer capacitance are associated with decreases in decay length.^20,21^ While we expect ionic liquids to deviate heavily from dilute electrolyte theory, it is still unexpected that the overall spatial extent of the diffuse double layer does not change while the capacitance increases significantly. This result suggests that interfacial capacitance is only minimally impacted by the diffuse double layer properties, indicating that the surface charge is well-compensated by the short-range structure.

Further, since electrostatic screening lengths are linked directly to bulk liquid properties, we note that minimal change between decay lengths in neat ionic liquids and a 1:1 mixture suggest that changes in bulk entropy do not influence long-range screening, suggesting that bulk entropy likely plays a minimal role on the observed changes in double layer capacitance. Rather, interfacial entropy within the short-range nanostructure must be the key driver toward enhancing capacitance.

We also determined that the confined ion layers present in neat [C_4_MPyrr]­[TFSI] exhibit an average effective thickness of 2.3 nm per surface under the highest compression tested. This 2.3 nm confined ion film is composed of approximately 4 ion layers per surface if ions are oriented along the shortest dimensions of the cations and anions (Figure S9). The confined ion layer thickness for neat [C_4_MIm]­[TFSI] was measured to be 1.1 nm per surface on average, which corresponds to approximately 3 ion layers per surface considering the smaller shortest dimension of the imidazolium cation. Notably, the mixture exhibits a confined ion layer thickness similar to [C_4_MIm]­[TFSI] measured by SFA, but more detailed interfacial evidence is provided by AFM measurements. We note that thicker bound ion layering revealed by SFA measurements of [C_4_MPyrr]­[TFSI] is corroborated by AFM measurements, which shows ion ordering extends further than what is measured in [C_4_MIm]­[TFSI] and the 1:1 mixture.

However, within the short-range nanostructure measured via AFM, we observe that the more disordered near-surface environment formed by the 1:1 [C_4_MPyrr]:[C_4_MIm] mixture compared to either neat ionic liquid is a direct manifestation of higher interfacial entropy in the mixture (Figure 6). Linking short-range AFM to EIS measurements (Figures 2 and 6), we attribute the increase in interfacial entropy to higher capacitance in ionic liquid mixtures. Observations of interfacial entropy are further corroborated by the slope of SFA force profiles approaching 1 mN m^–1^. We note that the force–distance profiles of the mixture near the point of closest approach exhibit sloping profiles (Figure 5c), which is in stark contrast to neat ionic liquids exhibiting sharp vertical force increases at similar separation distances (Figures 5a and b).

We also observed higher capacitance in size-mismatched anions with higher capacitance exhibited in mixtures of [C_4_MIm]­[BF_4_] and [C_4_MIm]­[TFSI] compared to either neat component. While the magnitude of increase in the anion mixtures is lower than the overall increase observed in cation mixtures, the common theme of increased capacitance in mixtures suggests that increased interfacial disorder is also likely playing a key role in mixed anion ionic liquids. While resolving the mechanistic details surrounding why anion mixtures exhibit smaller increases in double layer capacitance than cation mixtures is beyond the scope of our current study, we hypothesize that the large size disparity between the BF_4_ and TFSI anions may be a root cause for the similarity between the capacitance measured in anion mixtures and [C_4_MIm]­[BF_4_]. Since BF_4_ is such a compact anion compared to TFSI, the double layer formed in the anion mixture may exhibit much higher similarity to the neat [C_4_MIm]­[BF_4_] due to an interfacial depletion of the larger, conformationally flexible TFSI in anion mixtures.

Given that a 1:1 binary cation mixture induces a more disordered interfacial environment than the neat ionic liquids, we expect similar increased disorder in higher-component ternary cation mixtures as compared to neat ionic liquids. Indeed, our EIS measurements indicate that a 1:1:1 ternary cation mixture exhibits an increased capacitance over most of the voltage window as compared to any of the neat parent ionic liquids (Figure 4). Yet, we note that the increase in double layer capacitance for the ternary 1:1:1 cation mixture does not further increase beyond that measured for binary 1:1 cation mixtures. This suggests that transitioning from neat ionic liquids to binary cation mixtures leads to large changes to interfacial disorder and capacitance, while addition of further components to mixtures has a much smaller relative impact on interfacial structure and capacitance (Figures 3 and 4). Overall, our extensive investigations into mixtures with varied aromaticity and polarity suggest that mixing ionic liquids increases capacitance relative to neat components. This suggests that disordered, higher-entropy interfaces drive capacitance increases over changes in enthalpic contributions.

Future work aimed at determining the mechanism of increased capacitance in mixtures could be advanced by methods suited to characterizing single-nanometer distances away from surfaces under varying polarizations, such as electrochemical AFM.^53,65,66^ Additionally, the SFA measurements reported here were performed on mica surfaces at a single surface potential induced by potassium desorption into ionic liquids. Future SFA studies leveraging electrochemical control could yield additional insights into how entropy of mixing impacts double layer formation under the large polarization conditions of electrochemical devices.^67^ Nevertheless, we show that ionic liquid mixtures exhibit advantageous charge screening properties when compared to neat ionic liquids, which is promising for energy storage applications. Overall, the structural and electrochemical findings we report provide a framework for leveraging electric double layer entropy in correlated electrolytes for next-generation supercapacitors.

## Conclusions

We show how electric double layer disorder can be modulated by mixing ionic liquids to improve interfacial capacitance. Our results indicate that ionic liquid mixtures containing three or more distinct ionic species improve capacitance relative to neat ionic liquids over wide voltage ranges. Notably, co-ions and counterions both contribute significantly to charge screening, meaning that ionic liquid capacitance is dictated by both cations and anions at the interface regardless of electrode polarization. Further, we show that compositional entropy has a larger influence than ion size by revealing that mixture capacitance is systematically larger than neat ionic liquid capacitance, even when one ionic liquid contains large, nonpolar ions. Using surface forces measurements, we observe that diffuse layer decay lengths of a model ionic liquid mixture are similar to both neat ionic liquid components. Given that capacitance in the mixture is larger than in either neat ionic liquid, we conclude that the interfacial capacitance is largely decoupled from the diffuse layer properties. Further, we observe only minimal changes to the spatial extent of bound ion layers, with confined ion films of less than 2.3 nm per surface in all cases and the mixture exhibiting intermediate confined ion layer thickness between either neat component. Together, surface forces results suggest that changes to interfacial ion packing and disorder are critical to determining the interfacial capacitance in ionic liquid mixtures. Overall, our capacitance and surface structuring measurements provide avenues toward using double layer entropy to systematically design ionic liquid electrolytes for boosting the energy density of supercapacitors.

## Methods

### Chemicals and Purification

We purchased the ionic liquids [C_4_MIm]­[TFSI] (≥99% purity), [C_4_MPyrr]­[TFSI] (>99% purity), and [C_4_MIm]­[BF_4_] (>99% purity) from Iolitec. [C_4_MIm]­[TFSI], [C_4_MPyrr]­[TFSI], or [C_4_MIm]­[BF_4_] was diluted with ethyl acetate and treated with activated charcoal. The solution was stirred at room temperature for 48 h after which charcoal was removed via vacuum filtration. We passed the filtered solution through an activated alumina column using an ethyl acetate mobile phase. Using rotary evaporation, we removed the ethyl acetate solvent and dried the respective ionic liquids at 80 °C for 48 h under vacuum. [P_1444_]­[TFSI] (>98% purity) was purchased from Iolitec and dried for 48 h at 80 °C. After drying, all ionic liquids were colorless at room temperature.

We used a representative [C_4_MIm]­[TFSI] sample postdrying to confirm that water content was <50 ppm measured by a Mettler Toledo C10S Coulometric Karl Fischer Titrator. Ionic liquids were combined in appropriate molar quantities and dried at 80 °C under vacuum for 48 h to prepare mixtures. Chemicals were stored in a glovebox filled with industrial grade argon (Airgas) maintaining a dry environment of <0.5 ppm oxygen and <0.5 ppm water.

### Synthesis of [C_4_MIm]­[AOT]

The ionic liquid [C_4_MIm]­[AOT] was synthesized using an ion metathesis reaction reported previously.^68^ Equimolar quantities of [C_4_MIm] chloride (>99% purity, Iolitec) and sodium AOT (≥99% purity, Sigma-Aldrich) were dissolved in dichloromethane and stirred at room temperature for 48 h. As the reaction proceeded, [C_4_MIm]­[AOT] was formed along with a sodium chloride precipitate. Sodium chloride was then filtered from the solution using vacuum filtration. We removed the dichloromethane solvent from the filtrate via rotary evaporation. The resulting solution was diluted with ethyl acetate and centrifuged for 30 min at 7000 rpm to remove any trace amounts of sodium chloride precipitate. We extracted the supernatant after centrifuging and removed ethyl acetate from [C_4_MIm]­[AOT] using rotary evaporation. [C_4_MIm]­[AOT] was then dried under vacuum at 70 °C for 12 h after which a silver nitrate assay was performed to confirm that no detectable sodium chloride side product was present. To verify that no thermal decomposition occurs during the drying process, we also conducted thermogravimetric analysis (TGA) using a TGA TA Q500 to determine that the synthesized [C_4_MIm]­[AOT] is stable up to 225 °C (Figure S10).

We verified the identity of synthesized [C_4_MIm]­[AOT] by collecting ^1^H, ^13^C, and ^1^H–^13^C heteronuclear single quantum coherence NMR spectra using a 400 MHz NMR Bruker Avance spectrometer equipped with a BBFO probe (Figures S11–S14). The NMR sample was diluted with CDCl_3_ with TMS used as a shift reference. For C_4_MIm: ^1^H NMR (400 MHz, CDCl_3_) δ_H_ 9.36 (s, 1H, H5), 7.32 (t, 1H, H3), 7.22 (t, 1H, H2), 4.18 (t, 2H, H6), 4.03–3.82 (m, 8H, H10), 1.78 (p, 2H, H7), 1.34–1.17 (m, 18H, H8), 0.89 (t, 3H, H9). ^13^C NMR (101 MHz, CDCl_3_) δ_C_ 136.7 (C5), 122.5 (C3), 120.8 (C2), 48.7 (C6), 33.2 (C10), 31.1 (C7), 18.4 (C8), 12.4 (C9).

For AOT: ^1^H NMR (400 MHz, CDCl_3_) δ_H_ 4.07 (dd, 1H, H2), 4.03–3.81 (m, 7H, H12’’ and H20’’), 3.23–2.97 (m, 2H, H4’’), 1.55 (q, 1H, H13), 1.47 (t, 1H, H21), 1.41–1.12 (m, 18H, H14, H15, H16, H18, H22, H23, H24, and H26), 0.80 (m, 12H, H17, H19, H25, and H27). ^13^C NMR (101 MHz, CDCl_3_) δ_C_ 170.6 (C3), 168.2 (C5), 66.9–66.1 (m, C12 and C20), 61.0 (C2), 37.7–37.4 (m, C13 and C21), 33.2 (C4), 29.4–27.7 (m, C14, C15, C22, and C23), 22.7–21.8 (m, C16, C18, C24, and C26), 13.0 (d, C19 and C27), 10.0–9.7 (m, C17 and C25).

Note: Solvent peaks at δ_H_ 7.21 and δ_C_ 76.1. Impurity peaks at δ_H_ 2.42 and 4.73 likely from residual acetone and water, respectively.

### Electrochemical Setup

All electrochemical experiments were conducted using a Biologic SAS SP-300 potentiostat in an argon-filled glovebox. We used a custom-built electrochemical cell housing a three-electrode setup described previously^41^ with a silver/silver sulfide nonaqueous reference electrode designed by Lawrence Livermore National Laboratories^69^ and a platinum coil wire counter electrode. We soldered a 100 nF capacitor to the reference electrode to bypass high electrode impedance. The ferrocene/ferrocenium redox pair was used to standardize our reference electrode. Glassy carbon (BASi, geometric surface area 0.071 cm^2^) or gold (BASi, geometric surface area 0.071 cm^2^) working electrode was used and mechanically polished exactly as detailed previously.^41^ Briefly, glassy carbon was polished mechanically using a 0.05 μm alumina slurry and gold was polished using a 1 μm diamond polish followed by 0.05 μm alumina slurry. After each electrochemical experiment, we held the working electrode at a potential of −1 V versus the reference electrode for 1 h to remove any contaminants that could not be removed using a mechanical polish.

### Electrolyte Stability and Capacitance Measurements

The electrochemical window of each ionic liquid electrolyte was confirmed using cyclic voltammetry at a 25 mV/s scan rate. For capacitance experiments, we first held the working electrode at OCP for 1 h. The starting OCP of the electrolyte systems ranged typically between −0.9 V to −0.2 V vs Fc/Fc^+^ and our capacitance measurements indicate that this shift in starting OCP does not meaningfully impact our interfacial capacitance trends and interpretation. Beginning at OCP, we used staircase potentio-electrochemical impedance spectroscopy to step the voltage in 50 mV increments in positive or negative directions until we reached the electrolyte stability limit. We applied a 10 mV sinus amplitude at each voltage and collected impedance over a 100 kHz to 1 Hz frequency range. Ten measurement points were conducted per decade, allowing us to probe 51 frequencies at each potential. After reaching the electrolyte stability limit, the electrochemical setup was refreshed with a polished working electrode and new electrolyte sample. We used this protocol because capacitive hysteresis is observed when beginning scans at either voltage limit, which lowers the accuracy of the measurement.^70^ We verified that beyond the negative or positive stability limit of each electrolyte system, the Cole–Cole model fails to fit EIS data (Figures S15 and S16). We confirmed that the voltage where the Cole–Cole model breaks down was consistent with the electrolyte stability windows determined using CVs. Repeated EIS experiments were conducted, which we report as averages with error represented within 95% confidence intervals. We reported error in both x and y (voltage and capacitance, respectively) to account for any variations in starting OCP, temperature fluctuations, and slight differences in electrode polishing. Capacitance at each voltage was extracted using the Cole–Cole analysis, which is detailed in the Supporting Information.

### Surface Forces Measurements

A μSFA (SurForce, LLC) was used to conduct surface forces measurements. For surface preparation, we cleaved single-crystalline mica surfaces ranging from 2 to 6 μm thickness. 45 nm of silver was deposited from 3.2 mm diameter silver pellets (≥99.99% purity, Thermo Scientific) onto one side of cleaved mica using an Edwards AUTO 306 Vacuum Coater. Mica surfaces were glued to glass discs using optical adhesive (Norland Products) with the silver side facing the glass. The surfaces were mounted into the μSFA with the mica sides facing one another in a cross-cylinder orientation. The μSFA was placed onto a Nikon Eclipse Ti-2 microscope with white light entering through the top of the SFA. Transmitted light was passed into an Andor Shamrock 500i spectrometer connected to a Photometrics Prime 95B camera to measure white light interference patterns.

The absolute separation distance between the two surfaces was determined using white light interferometry.^48,50^ Prior to sample injection, the μSFA was purged with ultrahigh purity grade nitrogen (Airgas) for 30 min to dry the mica surfaces. We then injected a droplet of ionic liquid sample between the two mica surfaces and brought the surfaces within ∼300 nm separation distance. Using a motorized micrometer, we approached the surfaces at a velocity of 3.0 nm/s and then retracted back to ∼300 nm three times. Three approach-retraction experiments were performed at a similar velocity using a piezoelectric motor. The three force profiles measured continuously using the motorized micrometer and piezoelectric motor were similar, so we chose a representative force profile for decay length fitting, averaging, and error analysis. Further, we repeated this protocol of collecting force–distance measurements over three independent replicates, where fresh electrolyte and mica surfaces were used for each experiment. Using the piezoelectric actuator, we also conducted force–distance runs on [C_4_MIm]­[TFSI] at a slower approach velocity of ∼1.1 nm/s (Figure S17). Force–distance profiles measured at ∼1.1 nm/s approach rates agreed with long-range screening behavior measured at approach velocities of 3.0 nm/s.

We chose to evaluate all ionic liquid systems at identical approach rates, as similarities in electrostatic decay lengths despite a 50% variance in ionic liquid viscosities^71^ provides further evidence that the results are detecting electrostatic screening as opposed to differences in hydrodynamic coupling. Further, Lhermerout and Perkin show that hydrodynamic and electrostatic forces in nanoconfined ionic liquids are on similar orders of magnitude over the separation distances we measure using the SFA.^72^ However, they demonstrate that for approach rates ranging from 1–10 nm/s, hydrodynamic viscous forces can be calibrated for accurately using a linear distance dependence. Since our approach rates fall within this 1–10 nm/s range, we used a linear distance dependence during force calibrations to effectively account for hydrodynamic forces so that decay length fitting only encompasses electrostatic contributions. For force calibration, we carefully selected distances that were outside the double layer regime but not far enough to include viscous forces to the measured double layer interactions. We have indicated the range over which we calibrate forces in representative [C_4_MIm]­[TFSI] force profiles for two different velocities measured using the SFA (Figure S18). In Figure S18, we also show the range over which velocities were estimated for each case.

The spring constant of the spring used to drive the surfaces together was calibrated to be 98 N/m. We used this spring constant to calculate the forces experienced by surfaces at each separation distance using Hooke’s law. Calculated forces were normalized by the radius of curvature of the dried mica surfaces. Force–distance profiles measured during each approach run exhibited a long-range exponential force decay. The exponential decay region was fitted using the linearized form of the Poisson–Boltzmann equation^34^ shown in eq 2:

2 $$\frac{F}{R}=\frac{8\pi e^{2}n^{*}{{(\kappa }^{*})}^{-1}}{k_{B}T}\frac{\Psi^{2}\left(1-\exp\left(-\kappa^{*}D\right)\right)}{\exp\left(\kappa^{*}D\right)-\exp\left(-\kappa^{*}D\right)}\approx \frac{8\pi e^{2}n^{*}{{(\kappa }^{*})}^{-1}}{k_{B}T}\Psi^{2}\exp\left(-\kappa^{*}D\right)$$

where e is the elementary charge, n* (meters^–3^) is number density of charge carriers, k_B_ (joule/kelvin) is the Boltzmann constant, T (kelvin) is temperature, Ψ (volts) is surface potential, (κ*)^−1^ (meters) is decay length, and D (meters) is separation distance. Here, Ψ and (κ*)^−1^ are fitting parameters, and decay length (κ*)^−1^ was extracted for each force profile collected. eq 2 models interaction forces between two cross-cylinder symmetric surfaces compressing a monovalent electrolyte system. The linearized Poisson–Boltzmann equation fits the asymptotic long-range decay of each ionic liquid electrolyte system, where the asymptotic portion of this fit appears linear on a semilog representation. At the asymptotic limit, long-range forces can be approximated by the simple exponential decay shown in eq (2). We averaged decay lengths from three force profiles measured during independent replicates conducted with fresh sets of mica surfaces and fresh electrolyte. Error is reported within 95% confidence for both the motorized micrometer and the piezoelectric motor, where error bars encompass three independent replicates to ensure statistical reliability of decay length measurements.

AFM measurements were performed using a Cypher ES AFM (Asylum Research, Oxford Instruments) with FS-1500AUD silicon tips (Oxford Instruments). Force measurements were collected in AC tapping mode with blueDrive photothermal excitation on freshly cleaved mica surfaces at room temperature. The spring constant of the AFM probe was determined in air using the thermal noise method^73^ (*k* = 19.2 ± 2 N/m) before submerging in each ionic liquid sample. The system was allowed to equilibrate for 1 h before measurement. We first performed large area scans (1 × 1 μm) to determine a defect-free region and then zoomed into a smaller area (20 × 20 nm) with atomic-scale smoothness. Force curves were collected over a vertical distance of 15 nm with a z-piezo velocity of 15 nm/s and a drive frequency of ∼ 300 kHz for tip oscillation.^74^ The measured force was obtained by multiplying the cantilever deflection value by the spring constant of the probe. The apparent separation from the surface was determined by shifting the z-piezo value by the slope in the hardwall region (taken as z-piezo values beyond the last observed force step) and applying a zero offset. The maximum applied force was 15 nN, and a minimum of 150 force curves were collected at multiple locations on the mica surface. These force curves were converted into heatmaps shown in the manuscript using bin sizes of 0.01 nm by 0.05 nN.

## Supplementary Material



## Notes

Cole–Cole analysis and data, cyclic voltammetry data, Nyquist plots, SFA force–distance profiles, closest SFA approach distances, AFM force–distance retraction heatmaps, capacitance versus voltage plots, ion dimensions, TGA of [C4MIm]­[AOT], and NMR of [C4MIm]­[AOT] (PDF)
