# Supplementary material for: Enhancing Capacitance by Increasing Electric Double Layer Disorder in Ionic Liquid Mixtures
Source: ACS Nano. 2026 Oct 19;20(43):29054–67. doi: 10.1021/acsnano.6c12577 (PMC13738729; doi:10.1021/acsnano.6c12577)
Supplement: Supplementary file 1 [file nn6c12577_si_001.pdf]

Enhancing Capacitance by Increasing Electric Double Layer Disorder in Ionic Liquid Mixtures

Hrishikesh Tupkar[1], Samuel G. Johnstone,[1] Sila Alemdar[1], Jack McAlpine[1], Jack Verich[1], Matthew A. Gebbie[1*]

[1]University of Wisconsin–Madison, Department of Chemical and Biological Engineering, Madison, WI 53706, United States

*Corresponding author

Capacitance Data Fitting. Impedance collected at each potential was converted to capacitance using the relationship

$$\hat{C}(\omega) = \frac{1}{j\omega\hat{Z}(\omega)} \quad (3)$$

where ω is angular frequency, $\hat{C}(\omega)$ is complex capacitance, j is the imaginary unit, and $\hat{Z}(\omega)$ is complex impedance. Using equation (3), the transformed capacitance data was represented on a complex capacitance plane, which plots the real and imaginary components of capacitance on the *x*- and *y*-axis respectively. Each semicircle on the complex capacitance plane represents a capacitive process occurring on a specific timescale. The complex capacitance spectrum extracted from each voltage was fitted using the empirical Cole-Cole equation[1]

$$\hat{C}(\omega) = \sum_i \frac{C_i}{1 + (i\omega\tau_i)^{\alpha_i}} + C_0 \quad (4)$$

where i is the number of semicircles on the complex capacitance plane, C_i is the individual capacitance contribution from each semicircle, τ_i is the component time constant, and α_i is a parameter that accounts for system nonidealities.

Cole-Cole fitting on each ionic liquid impedance spectrum revealed only two capacitive processes occurring on varying timescales (Fig S4), which are physically represented by an arc and a tail at lower frequencies. The arc was fit to reveal a semicircle corresponding to double layer capacitance, while the tail was attributed to slower capacitive phenomena that would result in a semicircle at lower frequencies than 1 Hz. This low-frequency region has been attributed previously to ion rearrangements or electrode restructuring in the innermost layer of the electric double layer. However, we note that this process does not impact double layer charging and therefore only the faster capacitive process is used for quantitative determination of interfacial capacitance. We used the semicircle associated with electric double layer formation to extract capacitance at each voltage step.

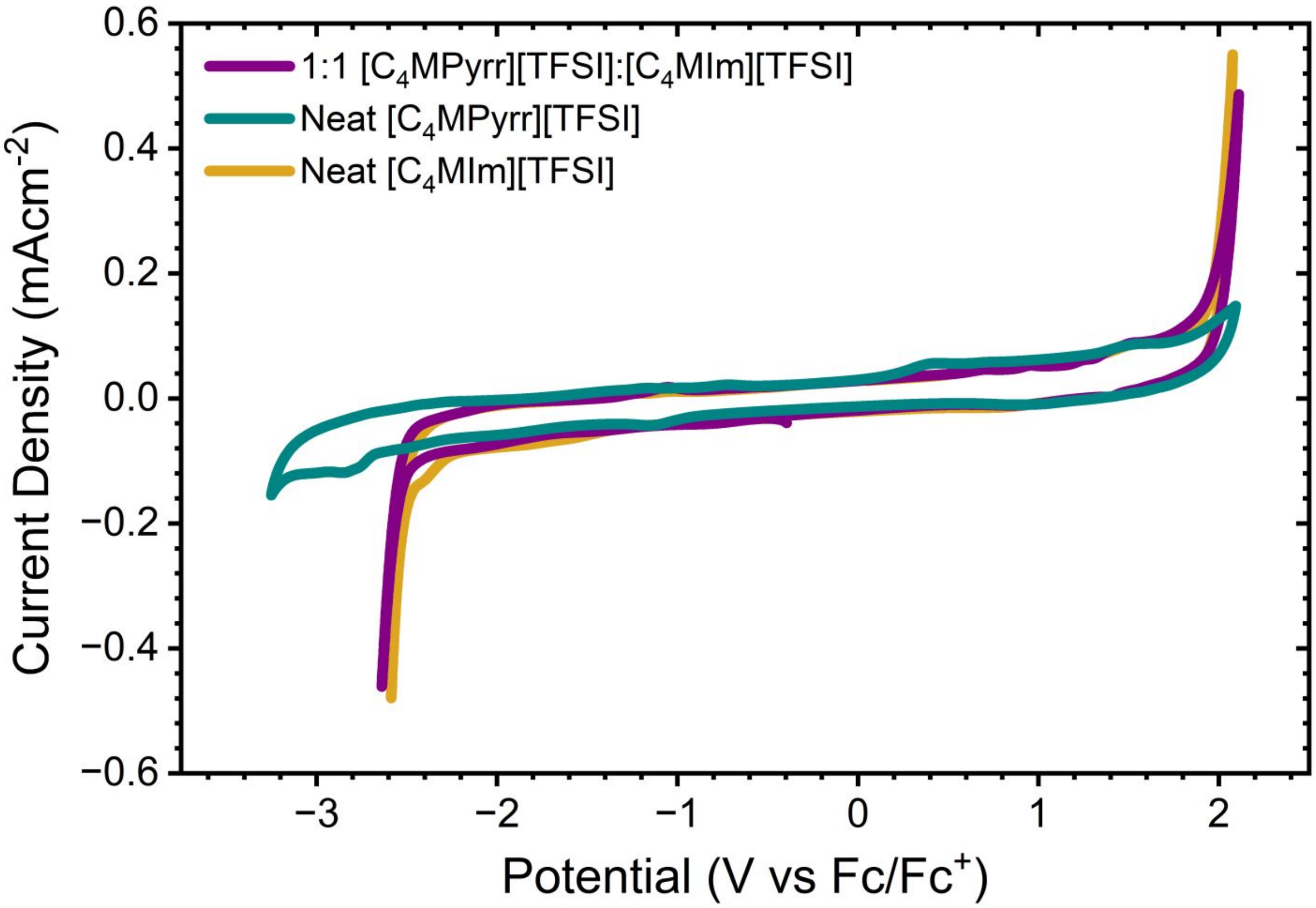

Figure S1. Representative cyclic voltammetry (CV) conducted on neat [C_4MIm][TFSI], neat [C_4MPyrr][TFSI], and the 1:1 [C_4MPyrr][TFSI]:[C_4MIm][TFSI] mixture at 25 mV/s scan rate (third cycle reported). CVs were conducted at a glassy carbon working electrode. All ionic liquid electrolytes were tested using these parameters to evaluate electrochemical stability.

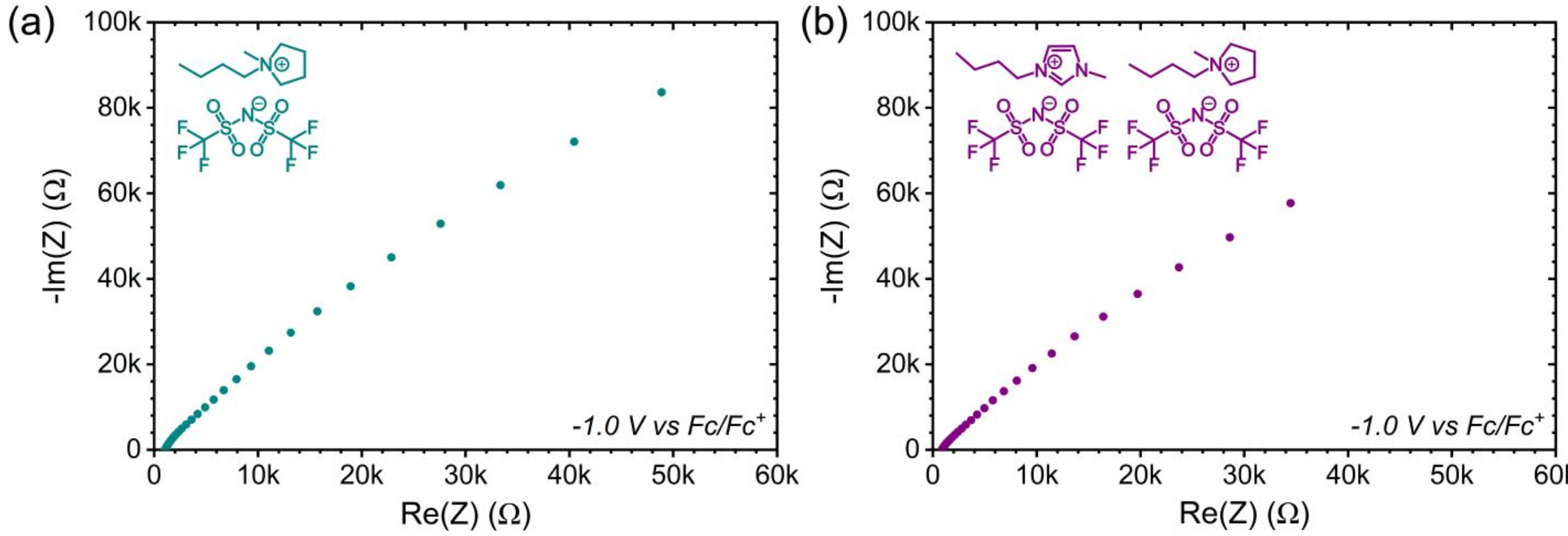

Figure S2. Nyquist representations of (a) Neat [C_4MPyrr][TFSI] and (b) 1:1 cation mixture [C_4MPyrr][TFSI]:[C_4MIm][TFSI] at -1.0 V vs Fc/Fc^+ on glassy carbon. We observe that capacitance at this potential is dictated by ion assembly from double layer formation. All ionic liquid electrolytes tested exhibited similar behavior on Nyquist plots.

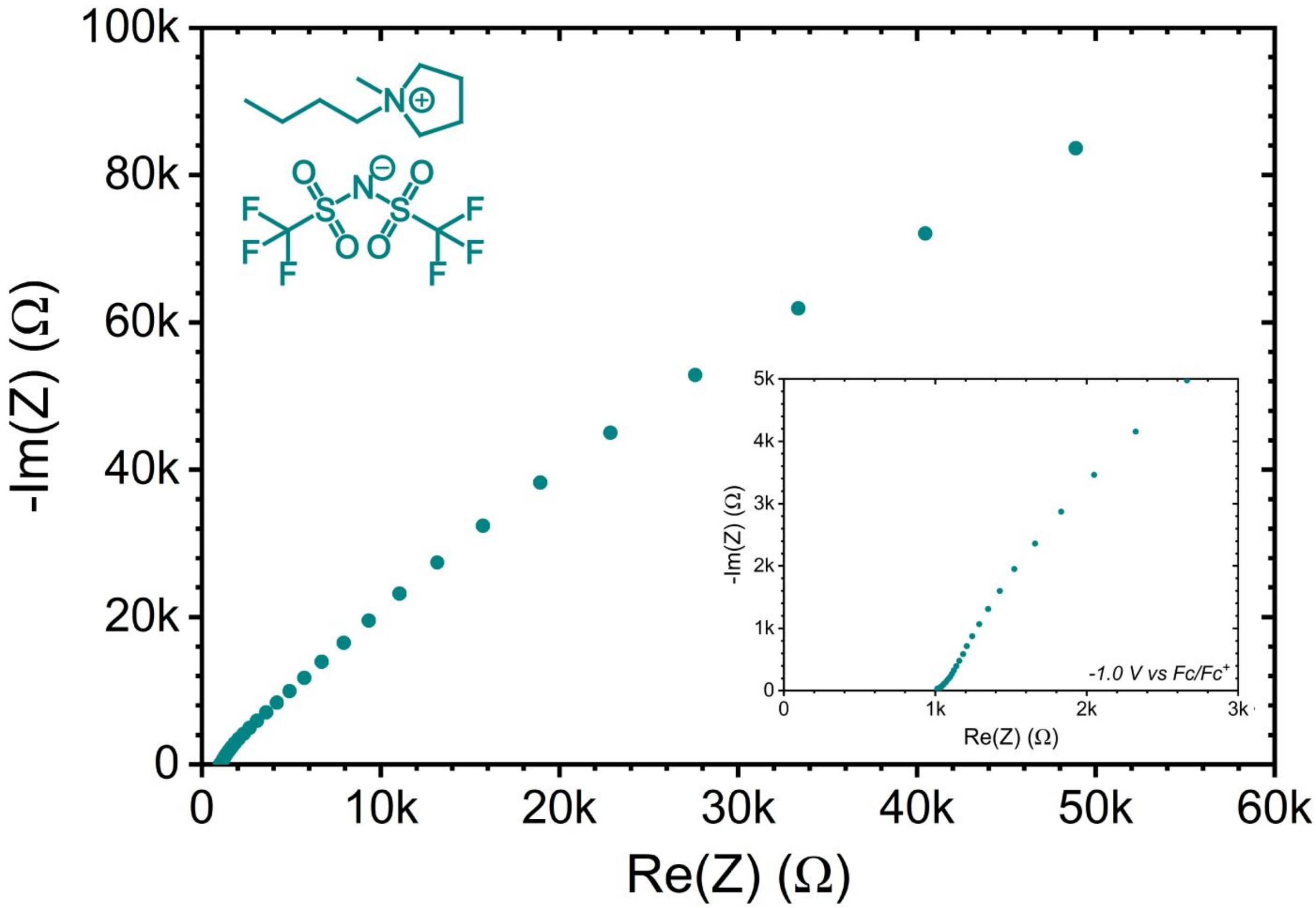

Figure S3. Nyquist representation of [C_4MPyrr][TFSI] EIS at -1.0 V vs Fc/Fc^+ with an inset focused on the high-frequency region.

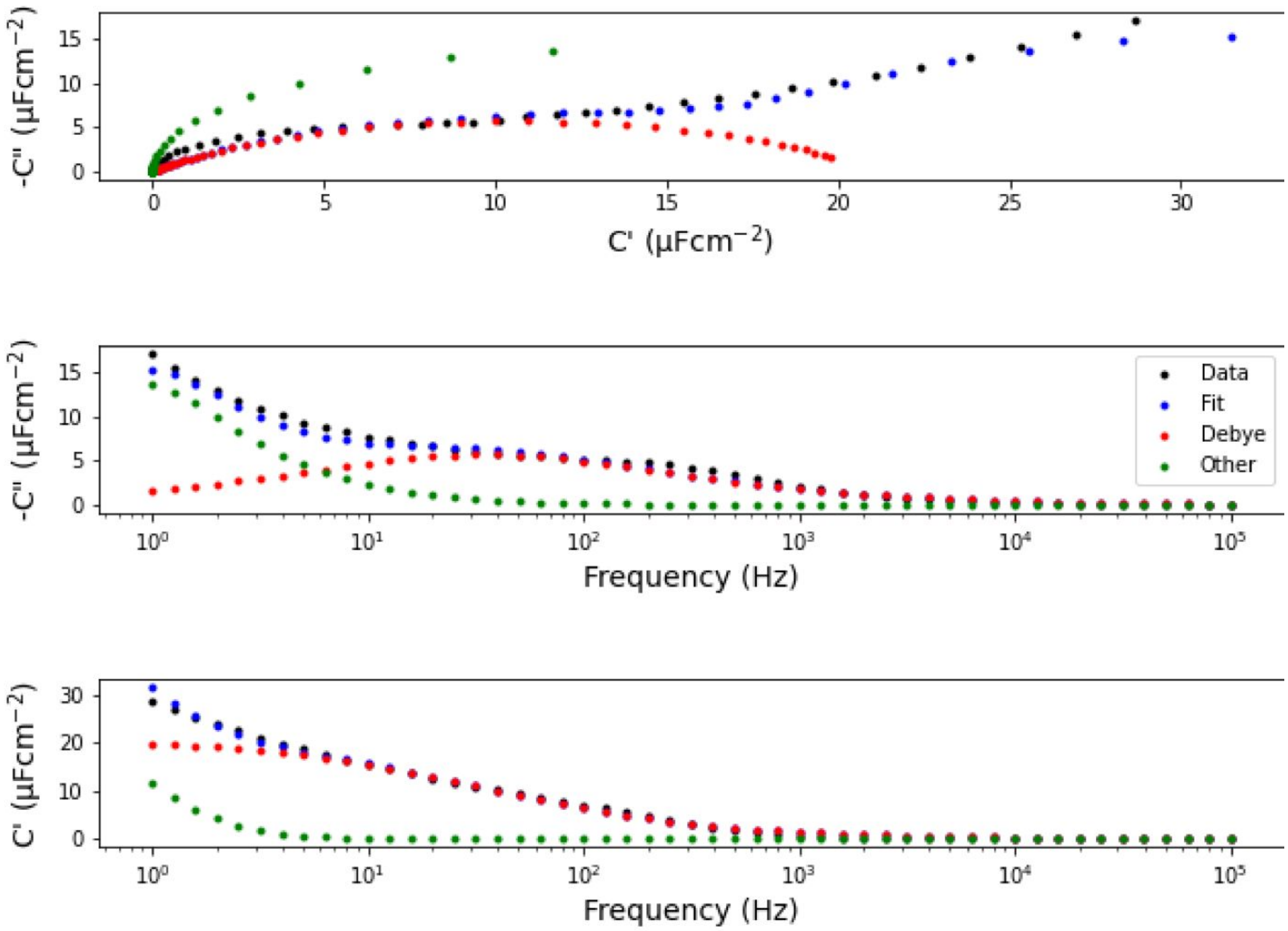

Figure S4. Cole-Cole analysis of 1:1 [C_4MPyrr][TFSI]:[C_4MIm][TFSI] cation mixture at -1.0 V vs Fc/Fc^+. Capacitance resulting from electric double layer structure was extracted from Cole-Cole fits.

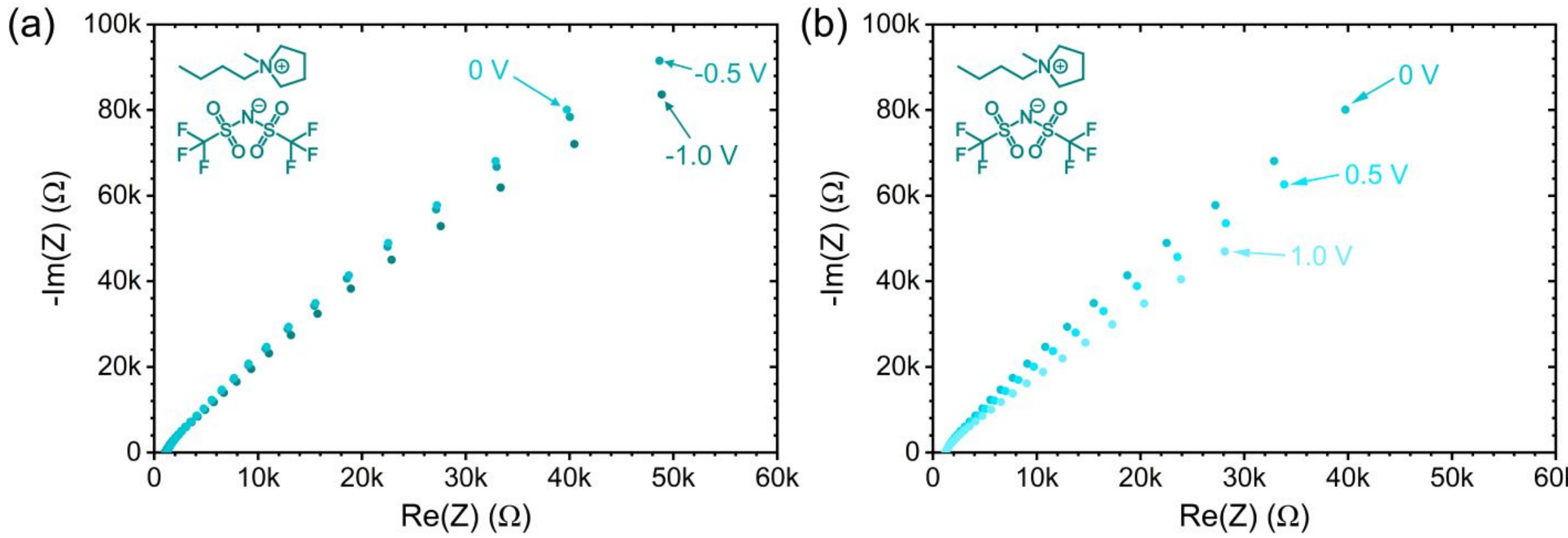

Figure S5. Nyquist representations of [C_4MPyrr][TFSI] at (a) negative and (b) positive potentials. Voltages are reported vs Fc/Fc^+.

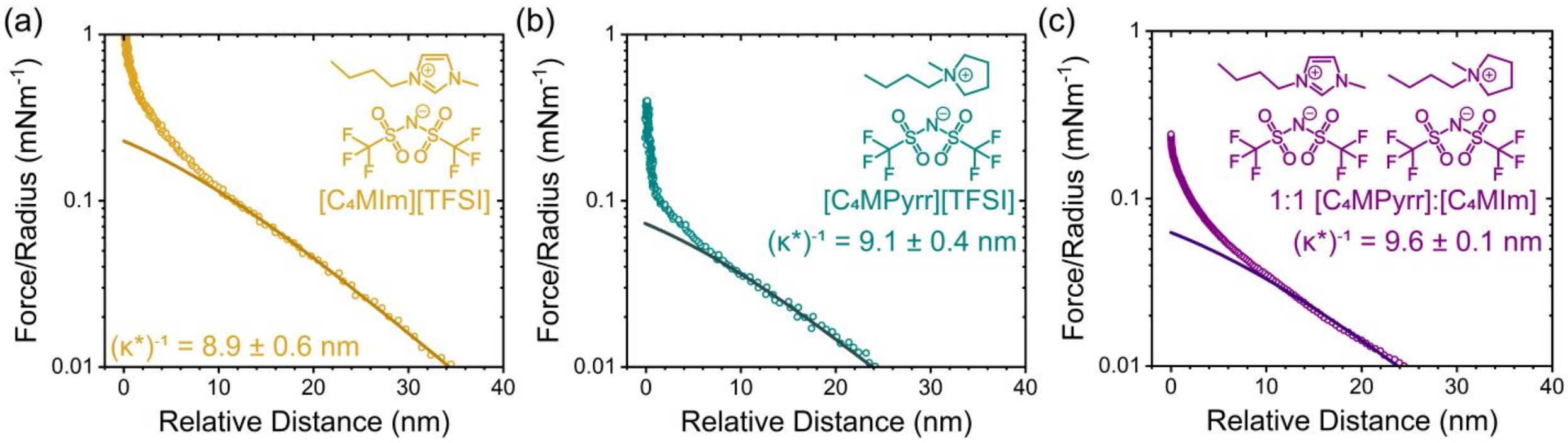

Figure S6. Representative force-distance profiles of (a) neat [C_4MIm][TFSI], (b) neat [C_4MPyrr][TFSI], and (c) 1:1 [C_4MPyrr][TFSI]:[C_4MIm][TFSI] measured using a piezoelectric motor. Using this motor, we primarily focused on measuring weak diffuse layer forces, so we observed inconsistencies in closest separation distances. Therefore, distances reported here are shifted relative to closest approach. We observe quantitative agreement in decay length fits with force curves measured using the motorized micrometer.

Table S1. Closest separation distances measured using the SFA motorized drive.

**Ionic Liquid Electrolyte**	Closest Approach (nm)	Distance Per Surface (nm)
Neat [C_4MIm][TFSI]	2.1 ± 0.4	1.1 ± 0.2
Neat [C_4MPyrr][TFSI]	4.5 ± 1.1	2.3 ± 0.6
1:1 [C_4MPyrr]:[C4MIm]	2.6 ± 1.1	1.3 ± 0.5

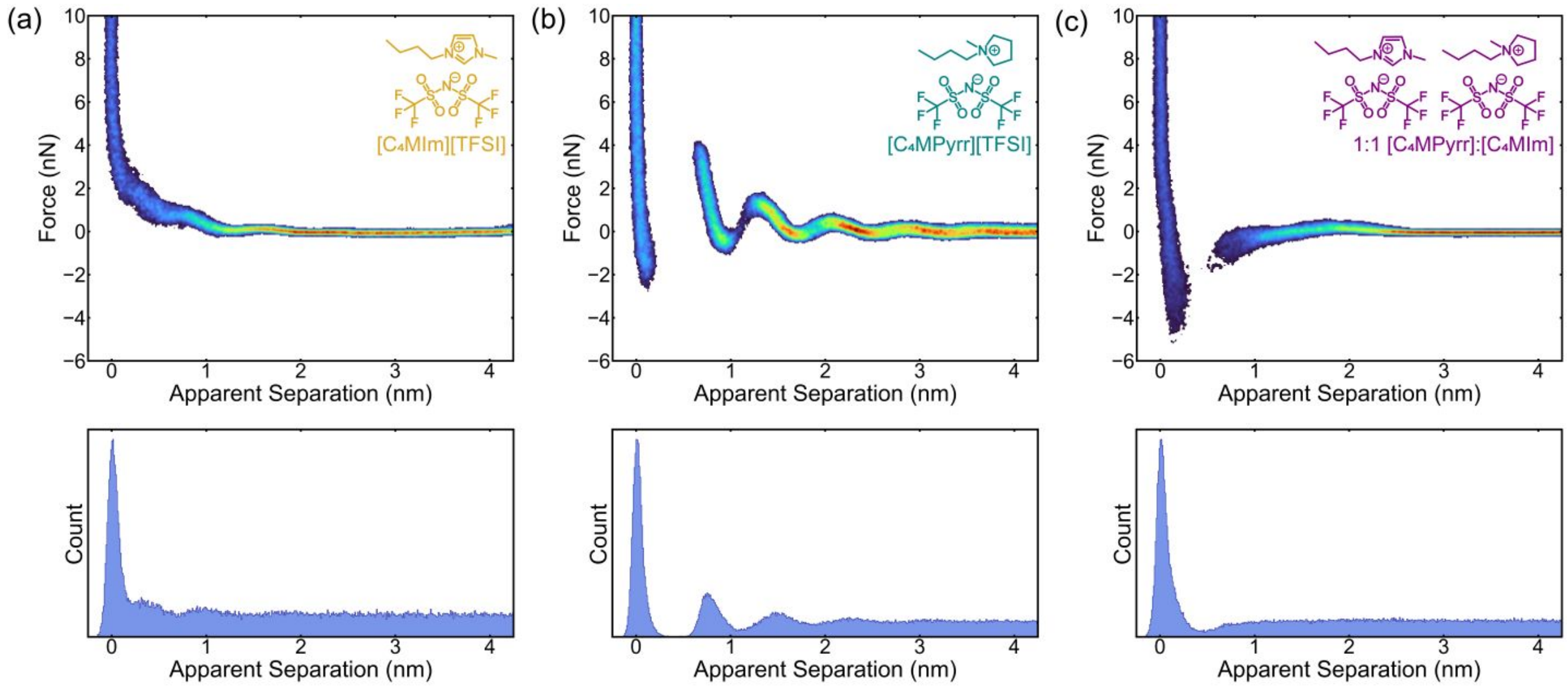

Figure S7. AFM force-distance heatmaps during retraction for (a) [C_4MIm][TFSI], (b) [C_4MPyrr][TFSI], and a (c) 1:1 [C_4MPyrr][TFSI]:[C_4MIm][TFSI] mixture on mica. Histograms constructed from each heatmap are pictured below force-distance profiles.

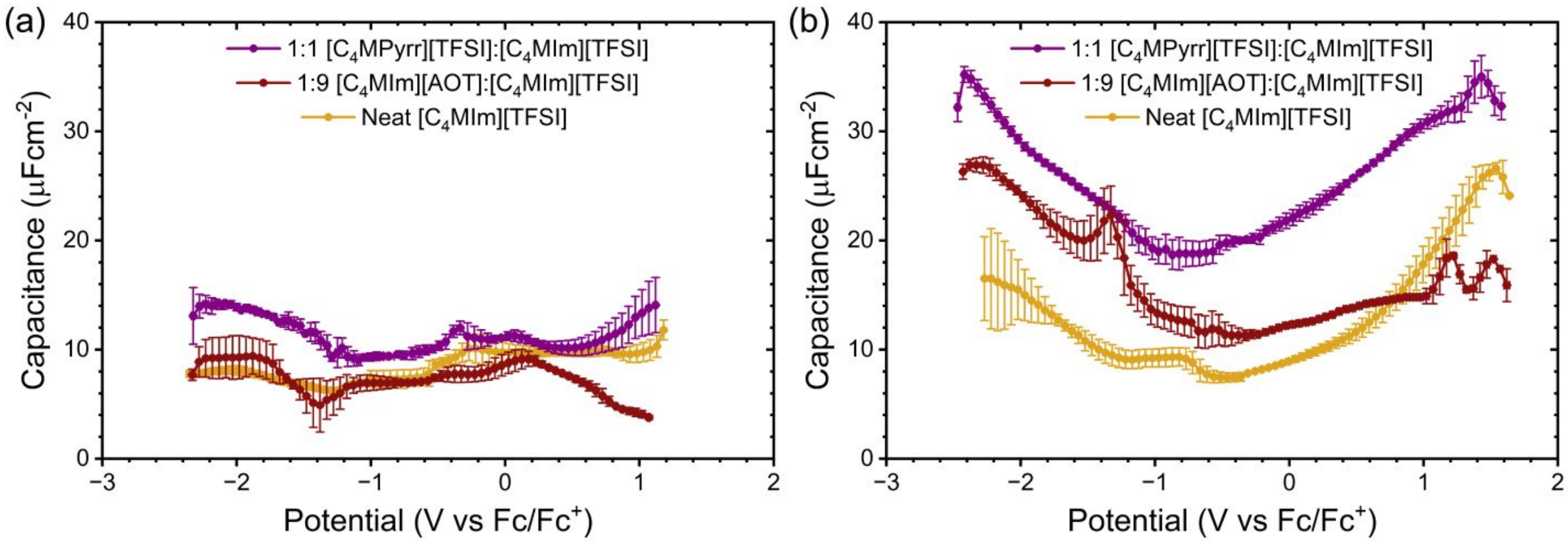

Figure S8. Potential-dependent capacitance of 1:1 [C_4MPyrr][TFSI]:[C_4MIm][TFSI], 1:9 [C_4MIm][AOT]:[C_4MIm][TFSI], and neat [C_4MIm][TFSI] on (a) gold and (b) glassy carbon working electrodes. While capacitance magnitudes are lower at the gold surface, we observe similar trends in capacitance between the two electrode surfaces.

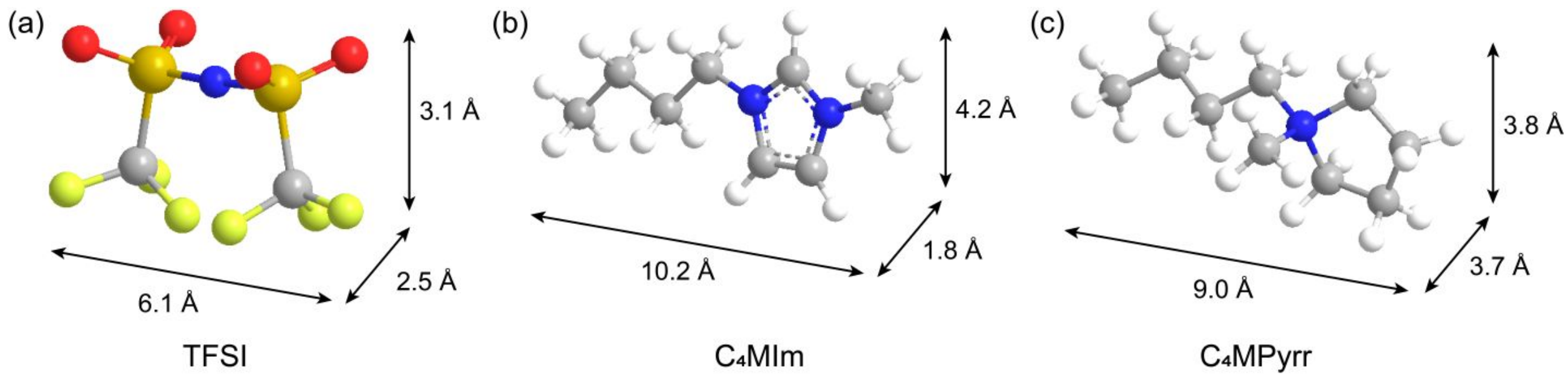

Figure S9. Ion dimensions for (a) TFSI, (b) C_4MIm, and (c) C_4MPyrr determined with MMFF94 energy minimization using the Chem3D software.

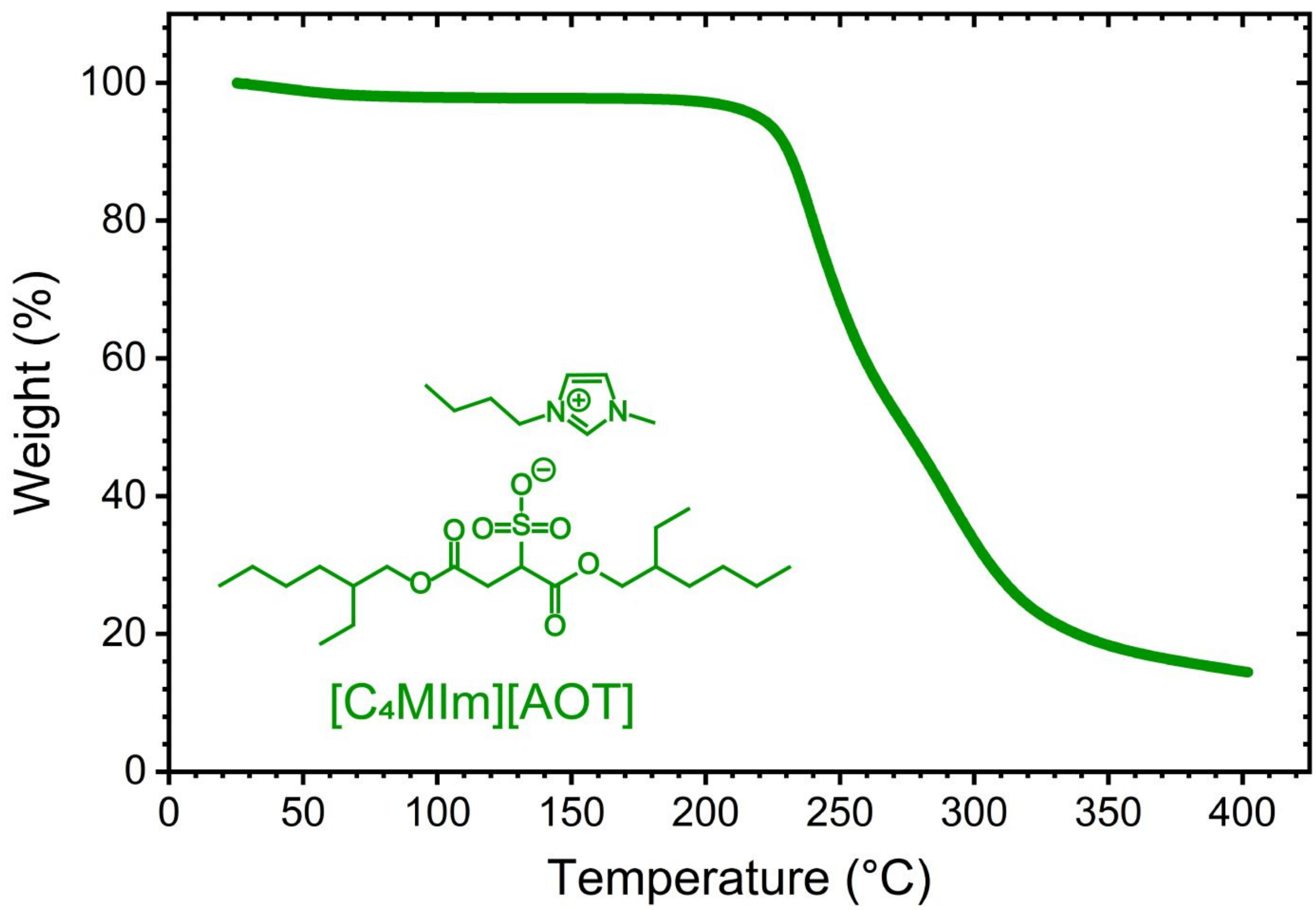

Figure S10. Thermogravimetric analysis of synthesized [C_4MIm][AOT] indicates thermal stability up to 225°C.

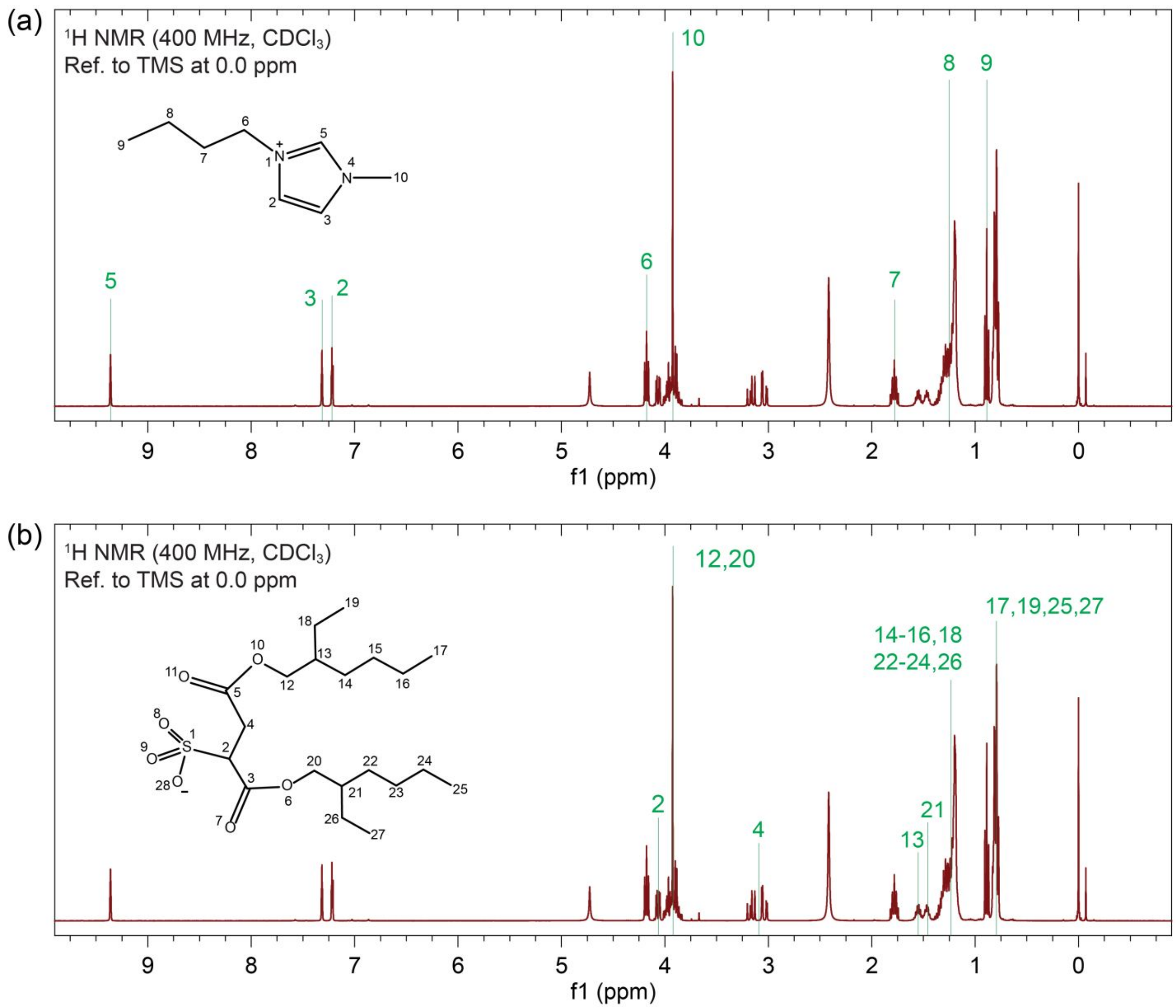

Figure S11. ^{1}H NMR of [C_4MIm][AOT] in $CDCl_3$ with peaks corresponding to (a) C_4MIm and (b) AOT identified.

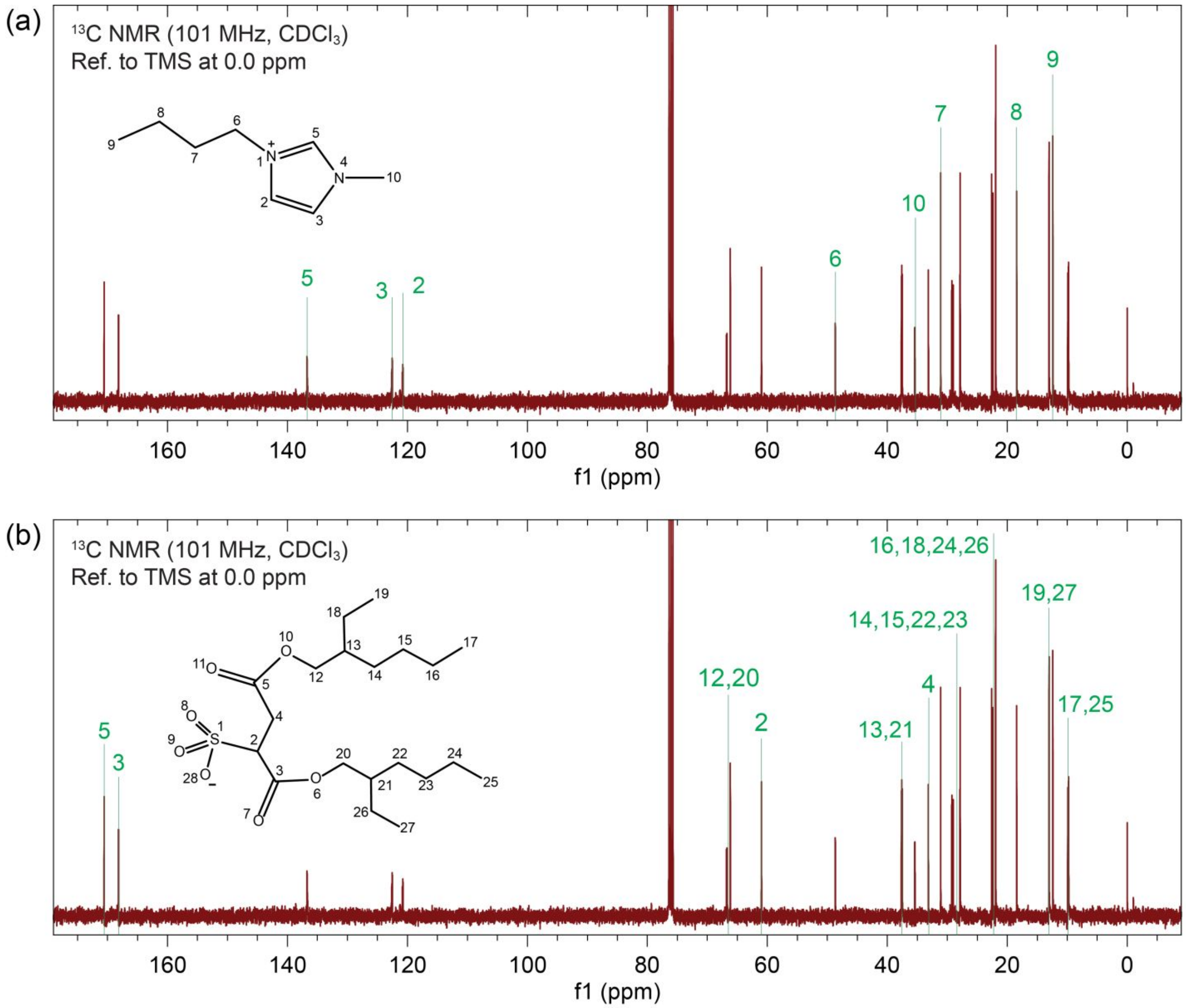

Figure S12. ^{13}C NMR of $[C_4MIm][AOT]$ in $CDCl_3$ with peaks corresponding to (a) C_4MIm and (b) AOT identified.

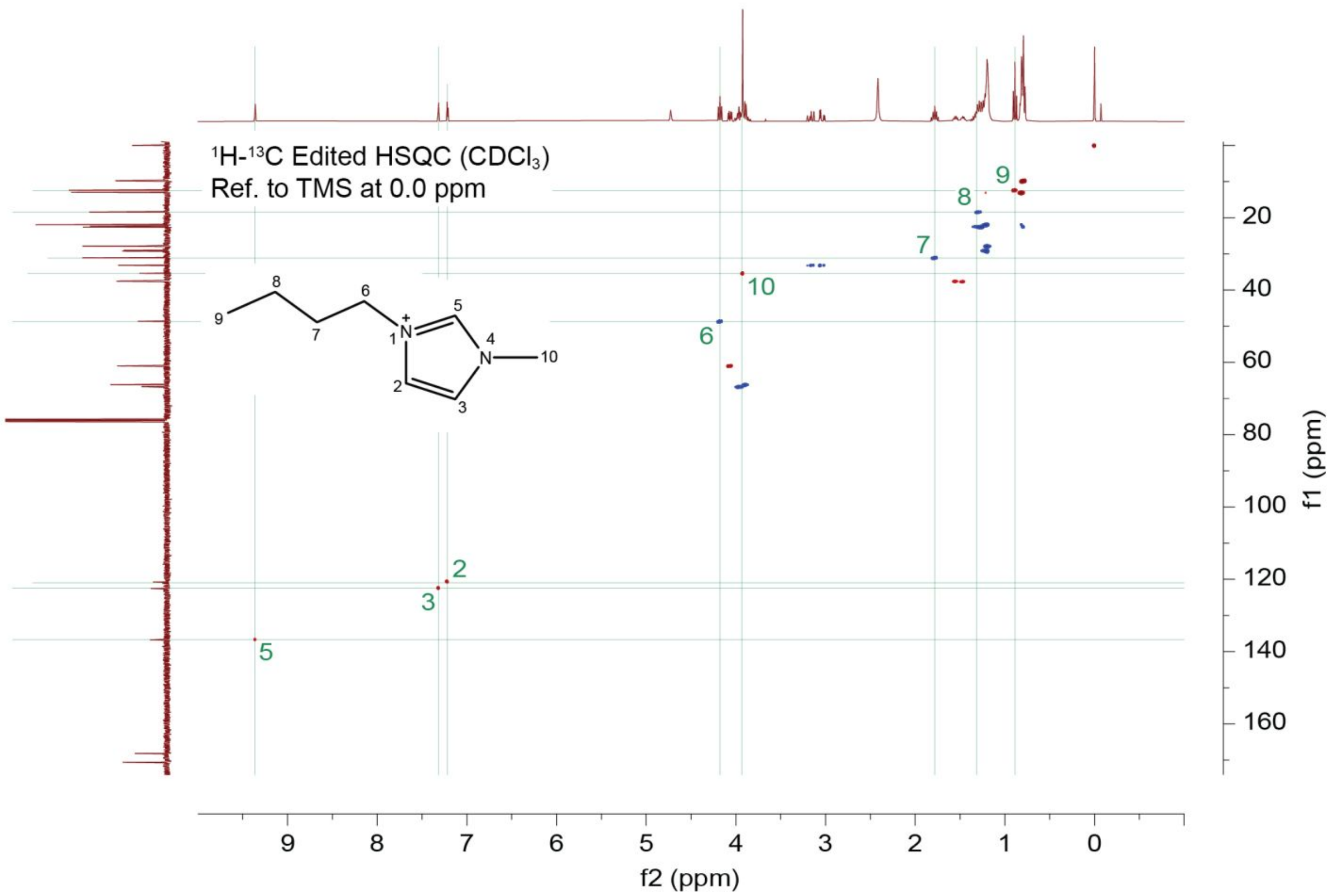

Figure S13. ^{1}H-^{13}C heteronuclear single quantum coherence (HSQC) NMR of [C_4MIm][AOT] in $CDCl_3$ with C_4MIm peaks identified.

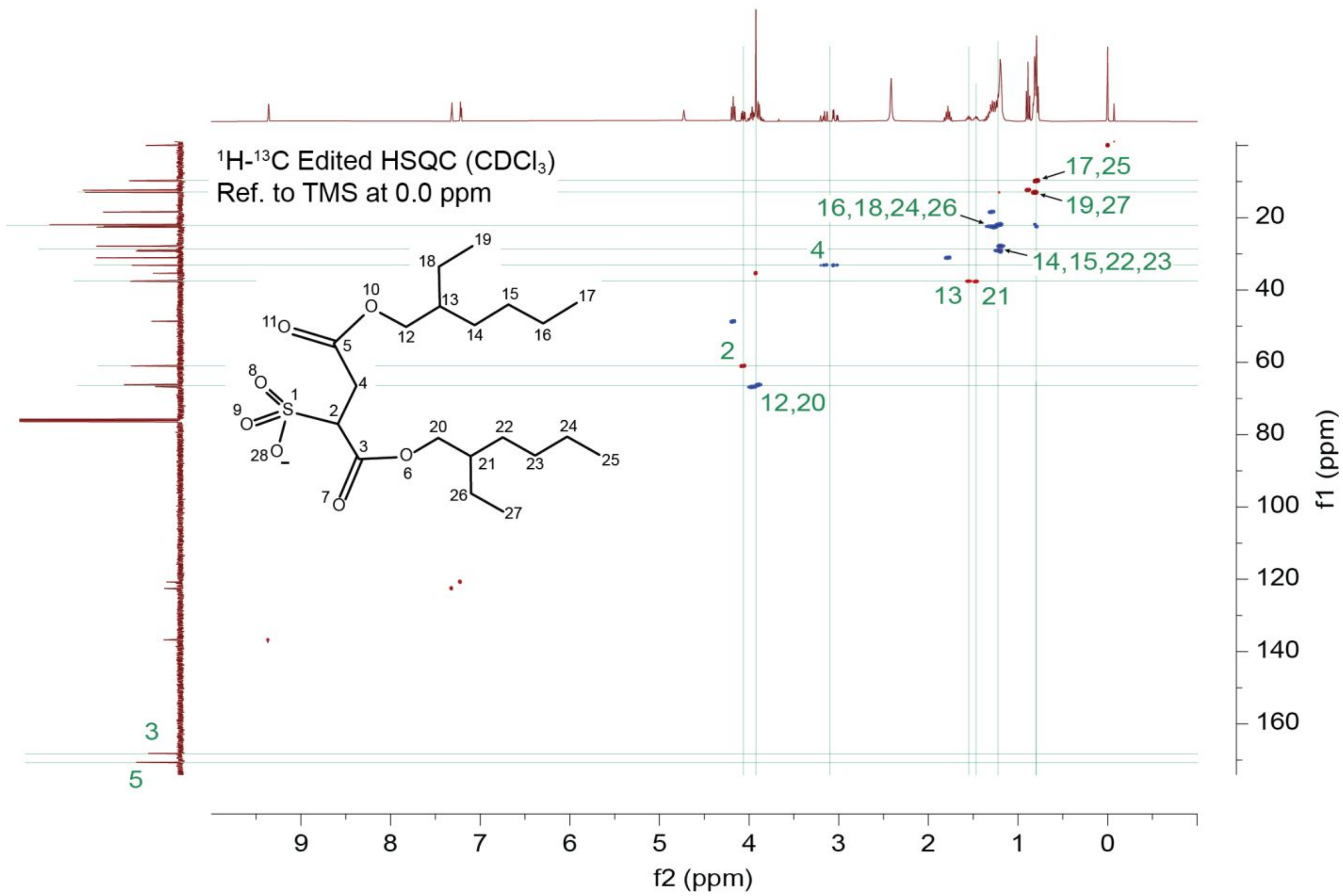

Figure S14. ^{1}H-^{13}C HSQC NMR of [C$_4$MIm][AOT] in $CDCl_3$ with AOT peaks identified.

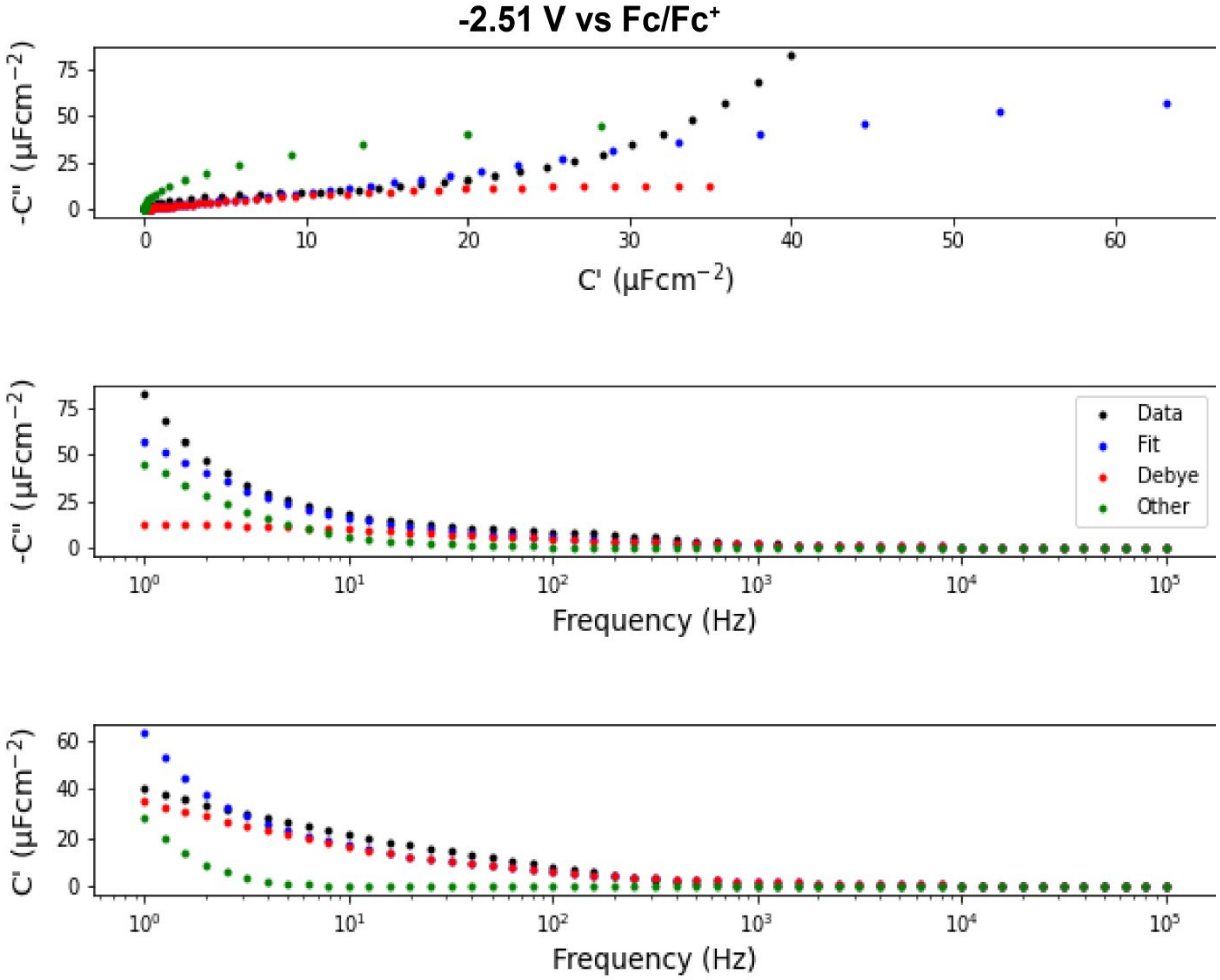

Figure S15. Cole-Cole analysis of 1:1 [C_4MPyrr]:[C_4MIm] at -2.51 V vs Fc/Fc^+ indicating breakdown past the negative stability limit.

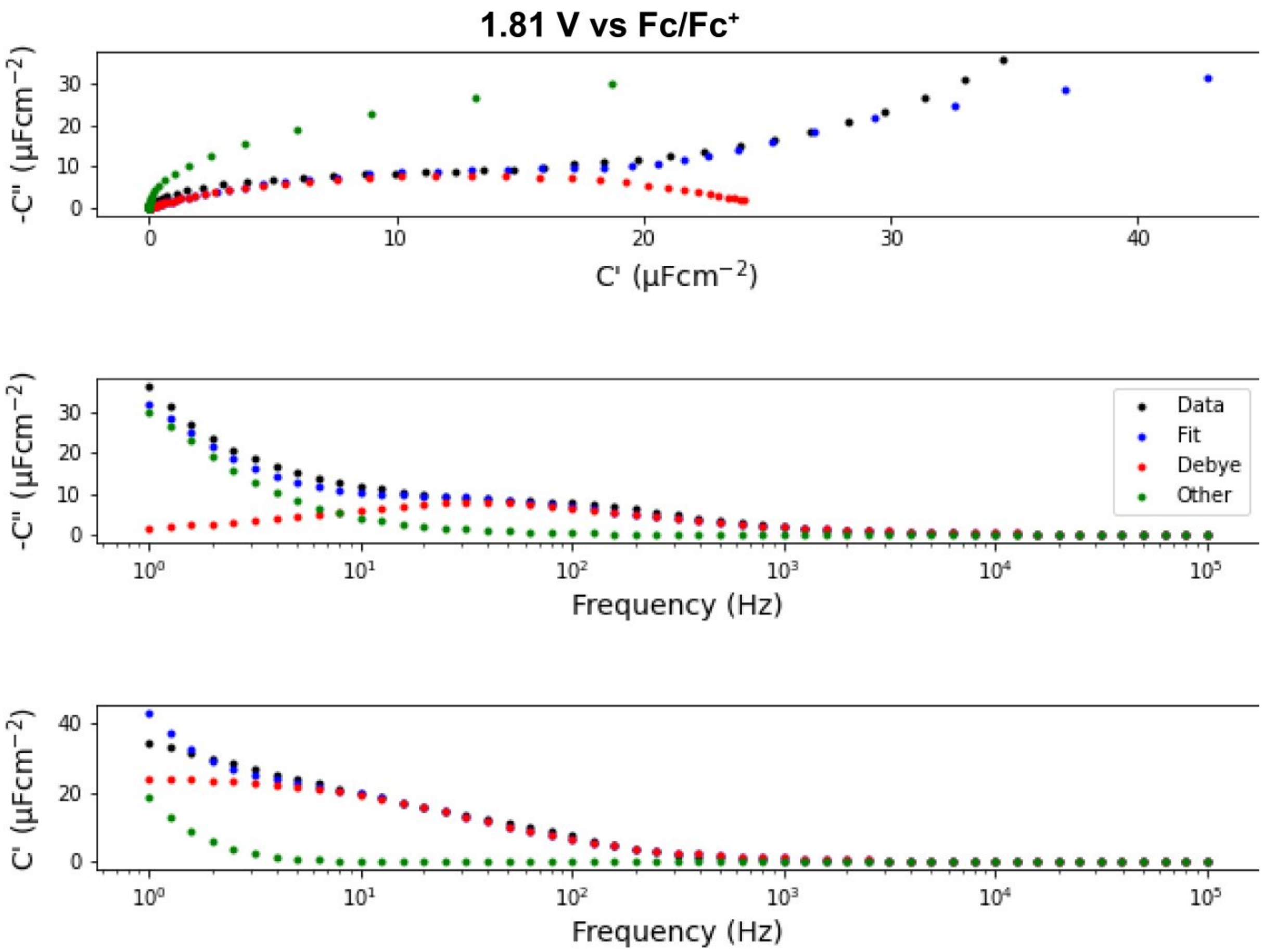

Figure S16. Cole-Cole analysis of 1:1 [C_4MPyrr]:[C_4MIm] at 1.81 V vs Fc/Fc^+ indicating breakdown past the positive stability limit.

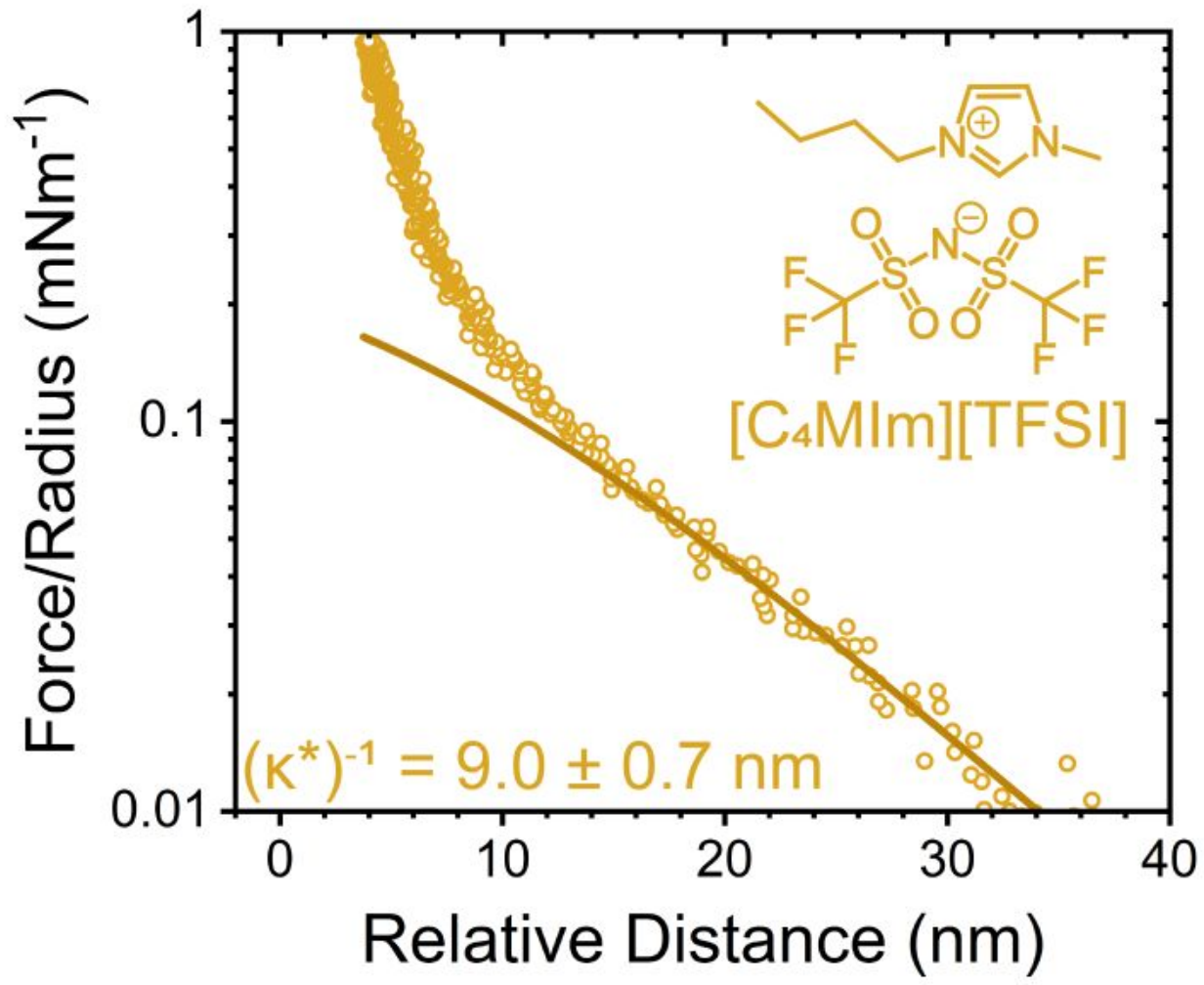

Figure S17. Representative force distance profile of [C_4MIm][TFSI] at ~1.1 nm/s approach velocity using a piezoelectric actuator.

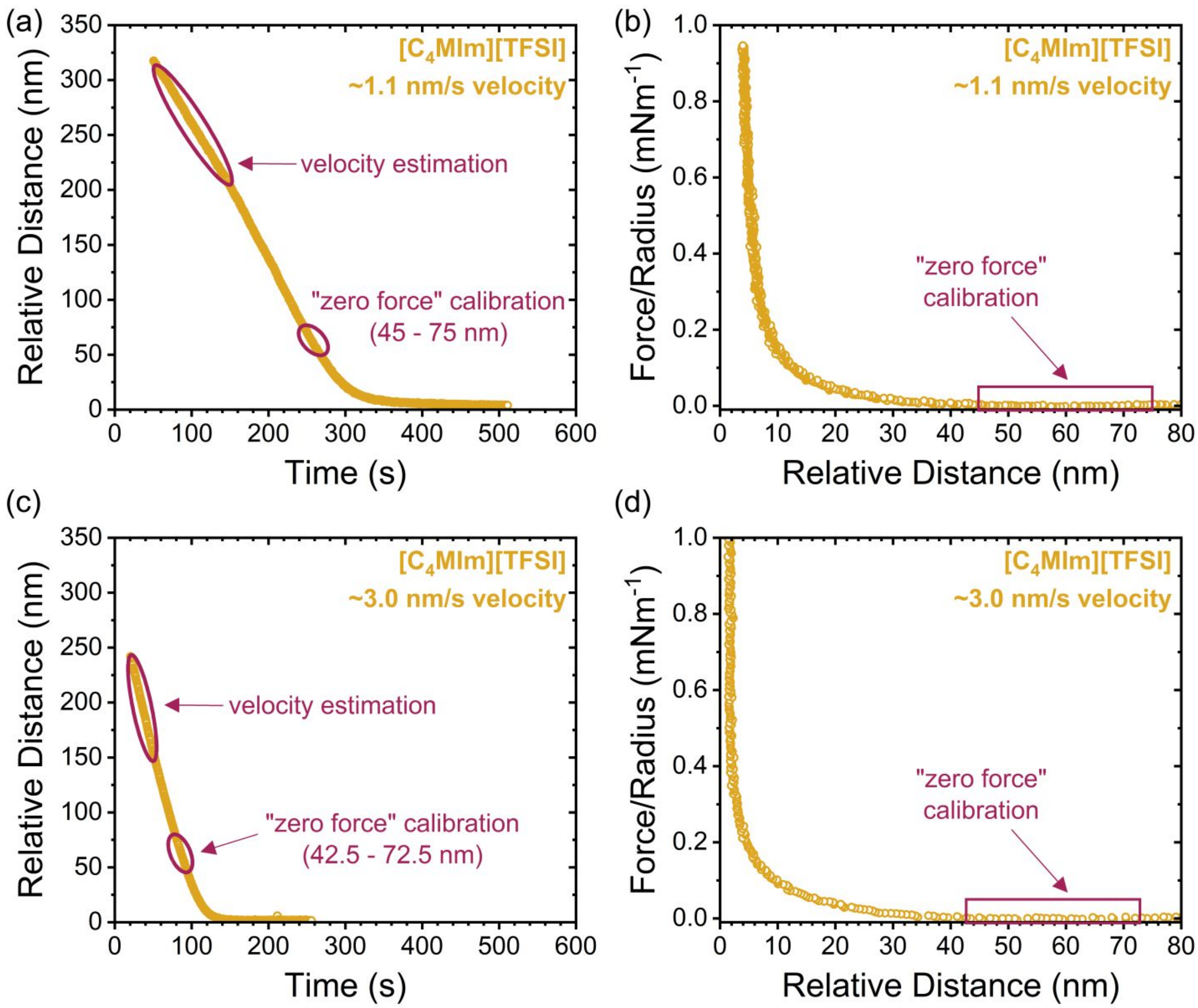

Figure S18. Representative SFA approach profiles showing (a) relative distance over time and (b) force-distance profiles at an approximate velocity of ~1.1 nm/s. Similar representative data is shown for profiles collected at ~3.0 nm/s in panels (c) and (d). The "zero" force calibration is indicated on each plot, with the region used to extract velocity indicated on the relative distance versus time plots.

References

(1) Endres, F.; Abbott, A.; MacFarlane, D. *Electrodeposition from ionic liquids*; Wiley-VCH, 2017.
